# Sulindac acetohydrazide derivative attenuates against cisplatin induced organ damage by modulation of antioxidant and inflammatory signaling pathways

**DOI:** 10.1038/s41598-022-15950-9

**Published:** 2022-07-11

**Authors:** Suhail Razak, Tayyaba Afsar, Nousheen Bibi, Mahmoud Abulmeaty, Mashooq Ahmad Bhat, Anam Inam, Janeen H. Trembley, Ali Almajwal, Maria Shabbir, Nawaf W. Alruwaili, Abdulrahman Algarni

**Affiliations:** 1 Department of Community Health Sciences, College of Applied Medical Sciences, King Saud University, Riyadh, Saudi Arabia; 2 Department of Bioinformatics, Shaheed Benazir Bhutto Women University, Peshawar, Khyber Pakhtunkhwa Pakistan; 3 Department of Pharmaceutical Chemistry, College of Pharmacy, King Saud University, Riyadh, 11451 Saudi Arabia; 4 Atta-Ur-Rahman School of Applied Biosciences, National University of Sciences and Technology, Islamabad, Pakistan; 5 Minneapolis VA Health Care System Research Service, Minneapolis, MN USA; 6 Department of Laboratory Medicine and Pathology, University of Minnesota, Minneapolis, MN USA; 7 Masonic Cancer Center, University of Minnesota, Minneapolis, MN USA; 8 Department of Medical Laboratory Technology, College of Applied Medical Sciences, Northern Border University, Arar, 91431 Saudi Arabia

**Keywords:** Biochemistry, Cancer, Computational biology and bioinformatics

## Abstract

This study aimed to explore the mechanisms of action of a sulindac acetohydrazide derivative, N'-(4-dimethylaminobenzylidene)-2-1-(4-(methylsulfinyl) benzylidene)-5-fluoro-2-methyl-1H-inden-3-yl) acetohydrazide, against anticancer drug cisplatin induced organ damage. Using a rodent model, various markers of organ function and signaling pathways were examined and validated by molecular docking studies. The study involves five groups of animals: control, DMSO, CDDP, CDDP + DMFM, and DMFM. Biochemical enzyme activity, histopathology, tissue antioxidant, and oxidative stress markers were examined. RT-PCR and western blot analyses were conducted for the expression of inducible cyclooxygenase enzyme (COX-2), nuclear factor kappa beta (NF-κB), p65, IL-1, TNF-α, and inducible nitric oxide synthase (iNOS). Flow cytometry analysis of CD4 + TNF-α, CD4 + COX-2, and CD4 + STAT-3 cells in whole blood was performed. Structural and dynamic behavior of DMFM upon binding with receptor molecule molecular docking and dynamic simulations were performed using bioinformatics tools and software. Treatment with DMFM reversed cisplatin-induced malondialdehyde (MDA) and nitric oxide (NO) induction, whereas the activity of glutathione peroxidase (GPx), and superoxide dismutase (SOD) in the kidney, heart, liver, and brain tissues were increased. DMFM administration normalized plasma levels of biochemical enzymes. We observed a marked decline in CD4 + STAT3, TNF-α, and COX2 cell populations in whole blood after treatment with DMFM. DMFM downregulated the expression factors related to inflammation at the mRNA and protein levels, i.e., IL-1, TNF-α, iNOS, NF-κB, STAT-3, and COX-2. Dynamic simulations and in silico docking data supports the experimental findings. Our experimental and in silico results illustrated that DMFM may affect protective action against cisplatin-induced brain, heart, liver, and kidney damage via reduction of inflammation and ROS.

## Introduction

The alkylating agent CDDP is an established and effective chemotherapeutic drug used in the treatment of several types of solid tumors. Nephrotoxicity, neurotoxicity, hepatotoxicity, and cardiotoxicity are dose-limiting side effects that often limit CDDP clinical use^1–3^. The ability to manage unwanted toxic side-effects of cisplatin is critically important for cancer therapy^4^, and thus various toxicity-inhibiting approaches are under investigation nowadays. CDDP interacts with cell machinery, including its well-characterized binding to deoxyribonucleic acid (DNA), forming covalent adducts with the bases adenine and guanine^5,6^. Although various mechanisms are proposed for the development of organ toxicities, the most frequently recognized and considered mechanisms are oxidative stress, apoptosis, and inflammation. Perturbation of Ca^2+^ homeostasis and structural and functional mitochondrial injury are other proposed modes of CDDP-induced toxic injury^7^.

As a metal-based drug, CDDP induces the development of free radicals, viz., the hydroxyl radical, hydrogen peroxide, and superoxide anion. The resulting oxidative stress is proposed to be a major mechanism of CDDP toxicity in cancer chemotherapy^8,9^. Both unresolved DNA damage and oxidative stress can activate cell apoptosis, which in turn results in the injury of tissue^10^. Inflammation involves a vast host of mediators, and the induction of pro-inflammatory genes, for example, COX-2 and inducible nitric oxide synthase (iNOS), might play a significant role in CDDP induced organ toxicities^11^. The pro-inflammatory cytokine TNF-α is involved in a large number of bioactivities, including immune response, proliferation, cell survival, cellular homeostasis, migration, and differentiation under normal physiological conditions^12^. Interleukin 1 (IL-1), is also a pro-inflammatory cytokine involved in numerous pathological and physiological activities and plays important roles in immune response, hematopoiesis, regulation of neuronal functions, and hepatic acute phase response^13^. The nuclear factor kappa B (NF-κB) transcription factor is a significant regulator of cell growth, apoptosis, and inflammation and is involved in the cellular response to stimuli such as free radicals, inflammatory cytokines, chemotherapeutics, carcinogens, and viral and bacterial agents. NF-κB is also crucial in the pathophysiology of numerous diseases involving inflammatory cell injury^14–16^. STAT3 is a pro-inflammatory transcription factor associated with tumorigenesis, including genetic alterations in several cancers^17,18^. STAT3 and NF-κB signaling are strongly linked^19,20^, with cross-regulatory interactions in a context-dependent manner. Various inflammation-associated genes targeted by NF-κB, such as interleukin- 6 (IL-6), encode important STAT3 activators; thus together, STAT3 and NF-κB co-regulate numerous inflammatory genes^14^.

Non-steroidal anti-inflammatory drugs (NSAIDs), recognized as pain relievers, function in part by inhibition of cyclooxygenases such as COX-1^21,22^. Sulindac is an indene derivative NSAID, which was identified to promote ulceration^23^. Novel sulindac derivatives have shown anti-inflammatory^24^ and anticancer activities^25–27^, function to inhibit COX-1^28^, and can activate PPARγ^29^. Synthesis of novel derivatives of NSAIDs has improved their safety profile which produced increased anti-inflammatory activity with a reduced ulcerogenic response. Recently a study revealed that a novel sulindac acetohydrazide derivative, N'-(4-dimethylaminobenzylidene)-2-1-(4-(methylsulfinyl) benzylidene)-5-fluoro-2-methyl-1H-inden-3-yl) acetohydrazide (DMFM), demonstrated highly significant anti-inflammatory and antioxidant activity as well as analgesic properties^23^. In the current study, we further evaluated the pharmacological potentials of DMFM and investigated the protective effects of Sulindac derivative “DMFM” against CDDP-induced cardiotoxicity, hepatotoxicity, neurotoxicity, and nephrotoxicity in rats. Several enzymatic and molecular biomarkers were analyzed to determine the mechanisms of action of DMFM against cisplatin-induced organ damage. Histopathological examinations were performed to detect any changes in organ morphology. The biological and physicochemical features of the DMFM compound were evaluated from the knowledge of its chemical structure and QSAR. Moreover, molecular docking studies were also executed to illustrate the molecular targets of DMFM against cisplatin organ damage.

## Methods

### Ethics statement

Before initiation of the study, approval was granted by Institutional Animal Care and Use Committee, Shaheed Benazir Bhutto Women University, Peshawar, Khyber Pakhtunkhwa, Pakistan. Studies described in the manuscript were performed in compliance with the ARRIVE guidelines.

### Animals

Adult male Wistar rats (n = 35; 12–14 weeks; 240–260 g) were provided by animal house Shaheed Benazir Bhutto Women University, Peshawar, Khyber Pakhtunkhwa, Pakistan. Pathogen-free housing conditions included a 12:12 h light: dark cycle, 25 °C, and 60 ± 10% relative humidity. The rats were fed a dietary formulation of fat (7.1%), protein (18.1%), fiber (15.5%), and carbohydrate (59.3%), 125, with water and food being provided ad libitum.

### Materials used

**Figure 1:**
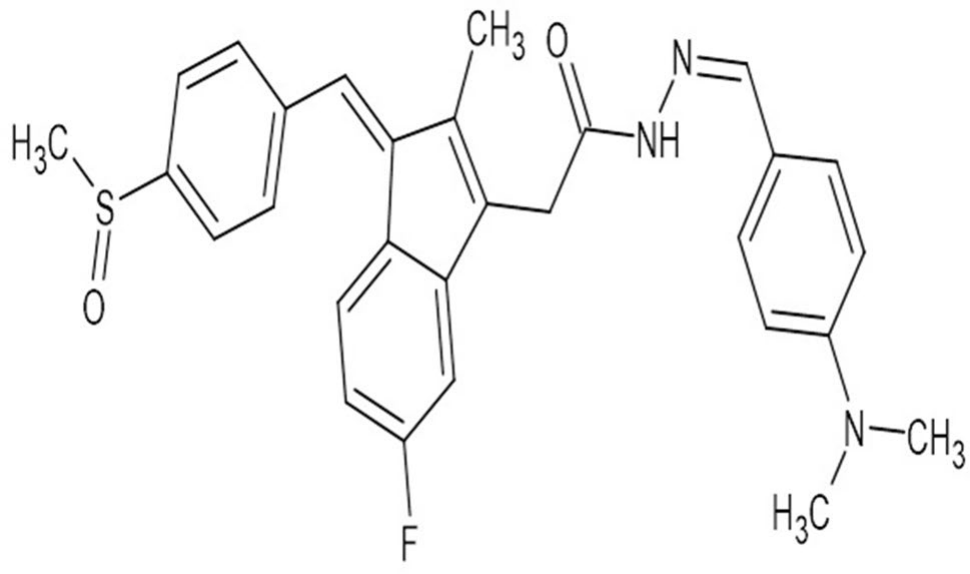
Structure of a Novel Sulindac derivative (DMFM).

The Sulindac derivative compound DMFM was synthesized by the Department of Pharmaceutical Chemistry, College of Pharmacy, King Saud University Saudi Arabia^23^. The structure of the compound is shown in Fig. 1.

### Acute toxicity testing of DMFM

Acute toxicity testing of DMFM was done following the guidelines of the Organization for Economic Cooperation and Development (OECD) for testing chemicals for acute oral toxicity^30,31^. Acute toxicity was monitored at 25, 50, 100, 200, 400, 800, 1600 and 2000 mg/kg body weight (BW). For determination of toxicity, observation was conducted individually for any neurological and behavioral changes such as tremors, convulsions, diarrhea, salivation, sleep, feeding, and lacrimation behavior as a sign of acute toxicity for 48 h^32^. DMFM was found to be safe up to 2000 mg/kg B.W dose and it did not persuade any lethal signs in rats like sedation, convulsions, diarrhea, and irritation. No mortality was observed up to 2000 mg/kg B.W dose.

### Dose standardization of DMFM

A pilot study was conducted for the selection of an effective dose with a protective effect against CDDP-induced multi-organ toxicity. Six groups having 3 rats in each group were taken. The dose of DMFM was selected as one‑tenth (200 mg/kg B.W) of the safe dose (2000 mg/kg B.W) from the acute toxicity study while 3 lowest doses (100, 50, and 25 mg/kg b.w) were also tested to find the lowest effective dose to be used for further experiments. 12 mg/kg B.w CDDP dose was selected to induce multi-organ toxicity. The dose of CDDP was selected based on published literature to induce organ toxicity^33,34^. DMFM doses were administered for 1 week and CDDP was administered on day 4. Both CDDP and DMFM were injected via the intraperitoneal route into rats in each group. After 72 h of CDDP administration, blood was collected via tail vein after restraining. Serum samples were analyzed for measuring various parameters (renal function test and liver function test). 25 mg/kg dose was selected for further experiments based on the data obtained from above testing (Supplementary Table 1).

### Drug administration and experimental design

The animals were categorized into five groups with seven animals in each group.

Saline (Group1) rats were given a single intraperitoneal (i.p.) injection of isotonic saline.

Vehicle control (Group 2) rats were given a single dose (i.p) of DMSO (1%).

CDDP (Group 3) rats were given a single dose (i.p) of CDDP (12 mg/kg).

CDDP + DMFM (Group 4) rats were given a single injection of CDDP (12 mg/kg) and 1 h later were given DMFM (25 mg/kg, i.p); DMFM injections continued for a total of 7 days.

DMFM (Group 5) rats were given DMFM (25 mg/kg, i.p) daily for a total of 7 days.

### Collection and preparation of samples

Rats were euthanized on the last day of the experiment by injecting a xylazine/ketamine mixture (2.5/75 mg/kg, respectively, i.p.)^35^. Anesthetized rats were secured in a supine position. The kidney, brain, liver, and heart were rapidly harvested with one part frozen in liquid nitrogen for RNA and protein analyses and the remaining tissue fixed in 10% formalin for histopathological studies. Blood was collected by cardiac puncture and a portion was used for flow cytometer analysis. The remainder of the blood was centrifuged for 15 min at 12,000 × g at 4 °C and the serum was removed for different biochemical measurements.

### Biochemical analysis of serum

Alanine aminotransferase (ALT), Alkaline phosphatase (ALP), Creatine kinase-MB (CK-MB), Creatine, Urea, aspartate aminotransferase (AST), and low-density lipoprotein (LDL) levels in serum were determined by AMP diagnostic kits following the instructions of the manufacturer.

### Oxidative stress markers and antioxidant status

SOD and GPX activity was measured as described^36–38^. NO activity was estimated by using MyBioSource kits (Catalog No: MBS 480,450).

### Measurement of malondialdehyde (MDA) and assessment of acetylcholinesterase (AChE) function in the brain

In the heart, liver, brain, and kidney tissue, the MDA levels were defined as an indication of lipid peroxidation as described^39^. Brain acetylcholinesterase activity was tested by the procedure described by Ellman et al.^40^.

### Protein extraction and immunoblot analysis

Western blot analysis was performed to check the protein expression of regulatory protein. 10 mg of tissue slice from each sample was mixed rapidly with ~ 600 μL of ice-cold lysis buffer (RIPA, Bio-Rad) supplemented with freshly added phosphatase and protease inhibitor cocktails 1:100 (Catalog Number PPC1010) and homogenize with an electric homogenizer, the blade was rinsed twice with another 2 × 200 μL lysis buffer, then maintain constant agitation for 2 h at 4 °C (eg place on an orbital shaker in the fridge). The homogenate was centrifuge for 20 min at 12,000 rpm at 4 °C in a micro centrifuge and supernatant was collected for protein quantification assay to quantify the protein concentration for each cell lysate ({Maddahi, 2011 #28}. Bradford assay was used to determine the protein concentration^41^. 30 µg protein sample was mixed with equal an equal volume 2X Laemmli sample buffer and samples were denatured at 100 °C for 5 min*.* Published protocols with minor alterations were used to conduct SDS-PAGE and immuno-blot examinations^42,43^. Protein samples were loaded for SDS-PAGE electrophoresis using the BIO-RAD Mini protein tetra system (10,025,025 REVA) and BIO-RAD Mini protein TGX precast gels. BIO-RAD trans blot Turbo_™_ transfer system was used to transfer protein to PVDF membranes (BIO-RAD). Membranes were blocked for 2 h with either 5% nonfat dry milk (BIO-RAD, Cat. #170-6404) or 5% BSA (Sigma) according to recommendations of antibody and incubated with primary antibody of interest at 4 °C overnight on shaker. Then the membranes were washed three times in TBS (Tris-buffered saline) and incubated with secondary antibody (eg HRP conjugated) diluted in blocking buffer for 1–3 h RT at the recommended concentration. β-actin antibody (1/1000 dilution in PBS/BSA/Tween20) was used as loading control. The antibodies were tested were: STAT3 (Thermofisher Catalog No. # MA1-13,042), COX2 (Invitrogen Catalog No. # PA1-9032), NFκB p65 (ThermoFisher Cat. # 2A12A7), TNF-α (Abcam Catalog No. # EPR19147), β-actin (Abcam Catalog No. # ab49900), and IL-1 (Abcam Catalog No. # ab2105). The antibody signals were identified by ECL western blotting substrate (BIO-RAD) and the BIO-RAD Gel documentation system (ChemiDoc MP System) was used for the blot visualization.

### RT-PCR analysis

The frozen kidney, heart, brain, and liver tissues were used to extract total RNA by following the company's guidelines (Promega Catalog No. # Z3101). The cDNA preparations were done with the Applied Biosystems™ High-capacity cDNA reverse transcription kit (Catalog No.#4,368,814). PCR reactions contained 10 µg cDNA, 10 µl FastStart Universal SYBR Green Master (Roche, Germany), 6 µM reverse primers, and RNAase free water in 20 µl total volume. The amplification and real-time analysis were done for 40 cycles with the following factors; 95 °C (10 min) to activate of FastStart Taq DNA polymerase; 60 °C (1 min) for amplification and real-time analysis^44^.

The gene expression was evaluated by 2^–ΔΔCT^. Table 1 contains the primers used.

**Table 1:** List of Primers.

Candidate gene	Primer
NF-κB p65	F: 5′- ACACCTCTGCATATAGCGGC-3′ R: 5′- GGTACCCCCAGAGACCTCAT-3′
TNF-α	F: 5′-GCGGAGTCCGGGCAGGTCTA-3′ R: 5′-GGGGGCTGGCTCTGTGAGGA-3′
COX-2	F: 5′-CACTCATGAGCAGTCCCCTC-3′ R: 5′-ACCCTGGTCGGTTTGATGTT-3′
IL-1	F: 5′-ACCTGCTCCACTGCCTTGCT-3′ R: 5′-GGTTGCCAAGCCTTATCGGA-3^/^
STAT-3	F: 5′-CCCCCGTACCTGAAGACCAAG-3′ R: 5′-TCCTCACATGGGGGAGGTAG-3′
iNOs	F: 5′-CTATGGCCGCTTTGATGTGC-3′ R: 5′-CAACCTTGGTGTTGAAGGCG-3′

### STAT3, COX-2, and TNF-α expression in total blood by flow cytometry analysis

Whole blood was collected by cardiac puncture for the investigation of STAT3, TNF-α, and COX-2 expression by flow cytometry as described^45^.

### Histopathologic assessment

Heart, kidney, brain, and liver tissues were placed for 24 h in 10% formalin and then embedded in paraffin. 4–6 µm sections from the blocks were stained with hematoxylin–eosin (H&E) and visualized using a Nikon Eclipse microscope (Ni Tokyo, Japan) at 40X magnification.

### Molecular configuration of receptors and ligand

The three-dimensional structures of rat TNF-α, NF-κB p65, SOD, GPX, STAT3, IL-1, and COX2 were predicted through homology modeling using the ITasser server (https://zhanglab.ccmb.med.umich.edu/I-TASSER) and the structure of iNOS was retrieved from Protein Databank (PDB) (http://www.rcsb.org) with PDB ID:4CX6. The structure of ligand N-(4-dimethylaminobenzylidene)-2-1-(4-(methylsulfinyl) benzylidene)-5-fluoro-2-methyl-1H-inden-3-yl) acetohydrazide (DMFM) was created in ChemDraw (www.cambridgesoft.com). Structure refinements and energy minimization were prepared using GROMMACS accessible in VEGA ZZ (http://www.ddl.unimi.it) and Chimera 1.5.6^46^.

### Molecular docking

Molecular docking of N-(4-dimethylaminobenzylidene)-2-1-(4-(methylsulfinyl)benzylidene)-5-fluoro-2-methyl-1H-inden-3-yl) acetohydrazide (DMFM) compound with selected target molecules iNOS, NF-κB p65, TNF-α, SOD, GPX, STAT3, COX2 and IL-1 were performed using AutoDock4.0^47^. Kollamen charges and polar hydrogen atoms were attached. Annealing factors for Van der Waals interfaces and hydrogen bonding were maintained at 2.5 A° and 4.0 A°. The total runs for individual docking testing were fixed to 100 with the default residual docking factors. Next, the finest docked complexes were carefully picked conferring to the binding free energy rate and molecular interfaces were elevated using UCSF chimera and Discovery Studio visualizer (http://accelrys.com/products/collaborative-science/biovia-discovery-studio)^47^.

### Molecular dynamic simulations

Molecular dynamics (MD) simulations tests were carried out to assess the structural behavior and stability of singular best-docked complex for separately candidate molecules. To evaluate the fluctuations as well as stability of protein C-alpha atoms, the time series of root mean square fluctuation (RMSF), as well as root, mean square deviation (RMSD) was assessed by GROMACS 4.5 package^48^. All systems were solvated by TIP4P water model^49^ tailed by the addition of Na^+^ and Cl^−^ counter ions to counterbalance all the systems. To the completion of simulations tests GROMACS tools and VMD^50^, PyMol (http://www.pymol.org) were used to scrutinize the fluctuation behavior and stability of all the systems.

### Pharmacokinetics investigation

Molecular descriptors and drug likeliness of DMFM compound were assessed by Molinspiration server (http://www.molinspiration.com), based on Lipinski Rules of five^51^. The AdmetSAR (http://www.admetexp.org) database was used to assess the absorption, distribution, metabolism, excretion, and toxicity of the compound. Osiris Property Explorer (http://www.organicchemistry.org/prog/peo/) was used to compute any objectionable toxic properties of the DMFM compound. Ligand Scout 3.0b (www.inteligand.com) was used to measure Pharmacophore features.

### Statistical analysis

All the experiments were carried out in triplicate. Results were expressed as mean ± standard error of the mean (SEM). One-way and two-way ANOVA were used for statistical analysis using Graphpad Prism software. *P* values of < 0.05 were considered significant.

### Ethical approval and consent to participate

The study protocol was approved by the ethics committee in the Shaheed Benazir Bhutto Women University, Peshawar, Khyber Pakhtunkhwa, Pakistan. Studies reported in the manuscript fully meet the criteria for animal studies specified in the ACS ethical Guidelines.

### Consent for publication

Not applicable.

## Results

Scheme 1 (Supplementary file S1) illustrates the preparation of the sulindac acetohydrazide derivative (DMFM) (Fig. 1). The previously developed protocol was used to synthesize DMFM^23^.

### Effect of DMFM treatment on CDDP induced alterations in rat organs

**Figure 2:**
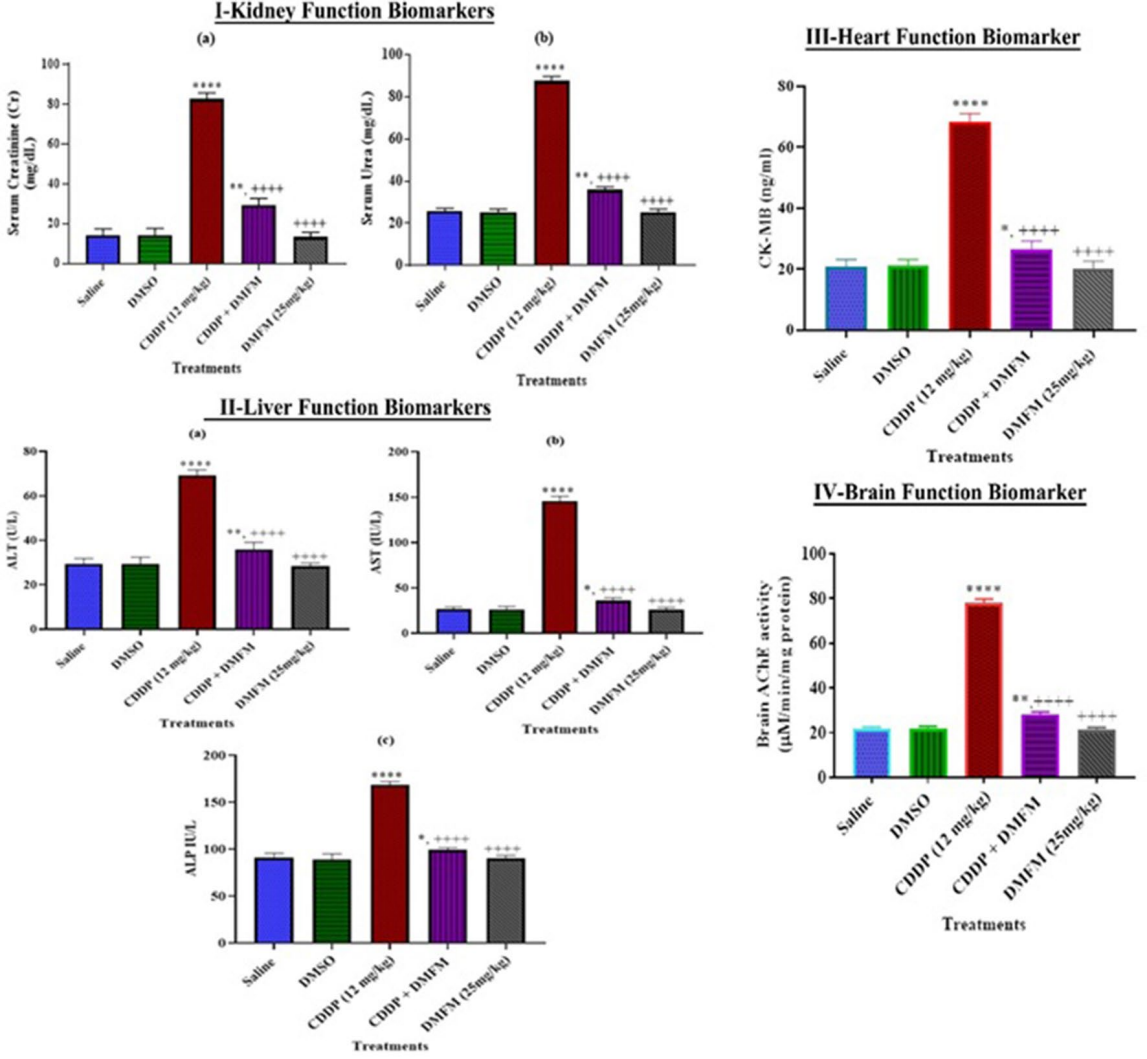
Effect of DMFM administration on organ injury biomarkers. Data presented as Mean ± SEM (*n* = 7). *, **** *p* < 0.05 and *P* < 0.0001 versus saline respectively, and ^++++^*P* < 0.0001 versus CDDP. Data were analyzed by One-way ANOVA combined with Tukey’s multiple comparison tests.

To evaluate how DMFM treatment potentially modulates the toxic side-effects of CDDP use, five groups of rats were treated. Established markers of organ function and injury were measured in the kidney, heart, liver, and brain. Biochemical analysis revealed high levels of creatinine and urea (*p* < 0.0001) in rats after CDDP treatment (single dose, 12 mg/kg, i.p). DMFM post-CDDP treatment considerably improved kidney functioning in CDDP exposed rats via lowering serum urea and creatinine levels within reference ranges (Fig. 2: Ia,b). Hepatocyte damage is the core cause of altered serum ALT, AST, and ALP levels. ALT, AST, and ALP were noticeably (*p* < 0.0001) elevated in CDDP exposed animals in contrast to the control group (Fig. 2: IIa,b,c); however, DMFM treatment after CDDP returned these serum markers to control levels. Serum levels for ALT, AST, and ALP following DMFM treatment alone were insignificantly different from the control group. The CK-MB isoenzyme is a blood biomarker indicating critical myocardial injury. As expected, acute CDDP exposure considerably elevated CK-MB presence in the blood. DMFM administration after CDDP injection considerably reduced the CK-MB secretion from the myocardium (Fig. 2: III). CDDP treatment caused considerable elevation of AChE activity in brain (78.75 ± 1.41 mM/min/mg protein), while DMFM reversed AChE induction considerably (27.13 ± 0.96 mM/min/mg protein, *p* < 0.0001, Fig. 2: IV).

### Effect of DMFM treatment on lipid profile

Total cholesterol, triglycerides, high-density lipoprotein (HDL), and low-density lipoprotein (LDL) cholesterol in plasma were evaluated (Table 2). Noteworthy variations in the blood lipid profile were observed in the CDDP treated group. The use of DMFM in CDDP administrated rats returned triglycerides, total cholesterol, and LDL to near control levels. DMFM treatment alone caused no notable change from control lipid profile levels.

**Table 2:** Effect of DMFM treatment on lipid profile.

Treatments	Total cholesterol (mg/dl)	HDL (mg/dl)	LDL (mg/dl)	TGAs (mg/dl)
Saline	70.50 ± 1.53	33.50 ± 0.75	44.95 ± 0.76	69.58 ± 0.98
DMSO vehicle	70.69 ± 1.58	33.59 ± 0.62	44.50 ± 0.83	70.01 ± 0.88
Cisplatin (12.5 mg/kg)	122.30 ± 2.42****	11.78 ± 0.93****	119.07 ± 2.35****	100.29 ± 2.28****
Cisplatin + DMFM( 12.5 + 25 mg/kg)	77.54 ± 1.11^++++^	29.07 ± 0.97^++++^	52.21 ± 0.78^++++^	79.29 ± 1.14*, ^++++^
DMFM (25 mg/kg)	69.01 ± 1.44^++++^	31.01 ± 0.81^++++^	41.76 ± 0.68^++++^	67.85 ± 1.03^++++^

Data are mean ± SEM, (*n* = 7). *, **** *p* < 0.05 and *P* < 0.0001 versus Control respectively, and ^++++^*P* < 0.0001 versus CP. Data analyzed by One-way ANOVA followed by Tukey’s multiple comparison tests.

### Effect of DMFM treatment on antioxidant and oxidative stress biomarkers

CDDP administration leads to a reduction in the activities of brush border membrane (BBM) and ROS scavenging enzymes^52^. Because CDDP administration is connected with oxidative trauma, the effect of DMFM on tissue NO and MDA content was studied. CDDP treatment significantly increased NO and MDA levels in tissues (*P* < 0.0001; Table 3). The use of DMFM reversed oxidative damage in CDDP-treated rat kidneys, liver, brain, and heart, and treatment with DMFM alone had no impact on oxidative indicator enzyme activity in these same tissues. Next, we observed a decrease in SOD and GPx activities in the kidney, liver, brain, and brain tissue of CDDP-treated rats (Table 4). DMFM treatment restored SOD and GPx activities following CDDP injection. Animals administered DMFM alone presented SOD and GPx enzyme levels similar to control in all observed organs, demonstrating a protective potential for DMFM administration.

**Table 3:** Effect of treatment with MMINA on oxidative stress in cisplatin-treated rat organs.

Treatments	MDA (ng/mg)				NO (ng/mg)			
	Liver	Kidney	Heart	Brain	Liver	Kidney	Heart	Brain
Saline	45.3 ± 2.11	34.65 ± 3.12	25.65 ± 2.15	32.79 ± 2.65	25.3 ± 1.11	31.65 ± 1.02	27.65 ± 1.17	19.79 ± 0.92
DMSO Vehicle	45.98 ± 2.34	35.01 ± 3.98	25.98 ± 2.01	32. 27 ± 2.34	25.98 ± 1.34	31.01 ± 0.98	27.98 ± 1.01	19. 97 ± 0.74
Cisplatin (12.5 mg/kg)	145.54 ± 3.76****	158.97 ± 4.87****	119.76 ± 5.21****	98.87 ± 3.11****	112.54 ± 2.71****	126.97 ± 3.37****	99.76 ± 5.21****	78.87 ± 1.61****
Cisplatin + DMFM (12.5 + 25 mg/kg)	56.87 ± 3.21*,^++++^	51.87 ± 3.11*,^++++^	49.43 ± 3.08*,^++++^	40.76 ± 1.98^++++^	36.67 ± 2.21*,^++++^	40.87 ± 2.30^++++^	34.43 ± 1.28^++++^	29.06 ± 0.99*^,++++^
DMFM (25 mg/kg)	42.12 ± 2.87^++++^	32.78 ± 2.54^++++^	23.76 ± 1.97^++++^	30.23 ± 1.76^++++^	22.12 ± 1.27^++++^	30.18 ± 0.99^++++^	25.76 ± 1.07^++++^	18.23 ± 0.76 ^++++^

Data are mean ± SEM, (n = 7). *, **** *p* < 0.05 and *P* < 0.0001 versus Control respectively, and ^++++^*P* < 0.0001 versus CP. Data analyzed by One-way ANOVA followed by Tukey’s multiple comparison tests.

**Table 4:** Effect of treatment with MMINA on antioxidant parameters in cisplatin-treated rat organs.

Treatments	SOD (U/g)				GPx (nM/min/g)			
	Liver	Kidney	Heart	Brain	Liver	Kidney	Heart	Brain
Saline	36.91 ± 0.49	48.67 ± 1.10	35.94 ± 0.90	30.96 ± 0.50	46.63 ± 1.00	56.15 ± 0.77	39.93 ± 0.54	40.51 ± 0.59
DMSO Vehicle	36.89 ± 0.75	48.55 ± 0.99	35.88 ± 0.89	30.34 ± 0.46	46.73 ± 0.95	56.21 ± 0.80	39.79 ± 0.59	40.29 ± 0.61
Cisplatin (12.5 mg/kg)	8.01 ± 0.55****	6.01 ± 0.50****	7.01 ± 0.51****	5.24 ± 0.06****	9.54 ± 0.27****	7.74 ± 0.49****	7.09 ± 0.71****	6.37 ± 0.57****
Cisplatin + DMFM(12.5 + 25 mg/kg)	29.99 ± 0.97^++++^	39.99 ± 1.00^++++^	28.74 ± 0.64^++++^	23.41 ± 0.49^++++^	39.12 ± 0.38^++++^	47.31 ± 0.59^++++^	31.98 ± 0.83^++++^	38.39 ± 0.66^++++^
DMFM (25 mg/kg)	38.00 ± 0.43^++++^	48.99 ± 1.09^++++^	36.03 ± 0.82^++++^	32.02 ± 0.87^++++^	47.06 ± 0.79^++++^	57.55 ± 0.61^++++^	41.06 ± 0.89^++++^	42.03 ± 0.79^++++^

Data are mean ± SEM, (n = 7). *, **** *p* < 0.05 and *P* < 0.0001 versus Control respectively, and ^++++^P < 0.0001 versus CP. Data analyzed by One-way ANOVA followed by Tukey’s multiple comparison tests.

### Effect of DMFM treatment on CD4^+^STAT3, CD4^+^TNF-α, and CD4^+^COX-2 T-cell populations

**Figure 3:**
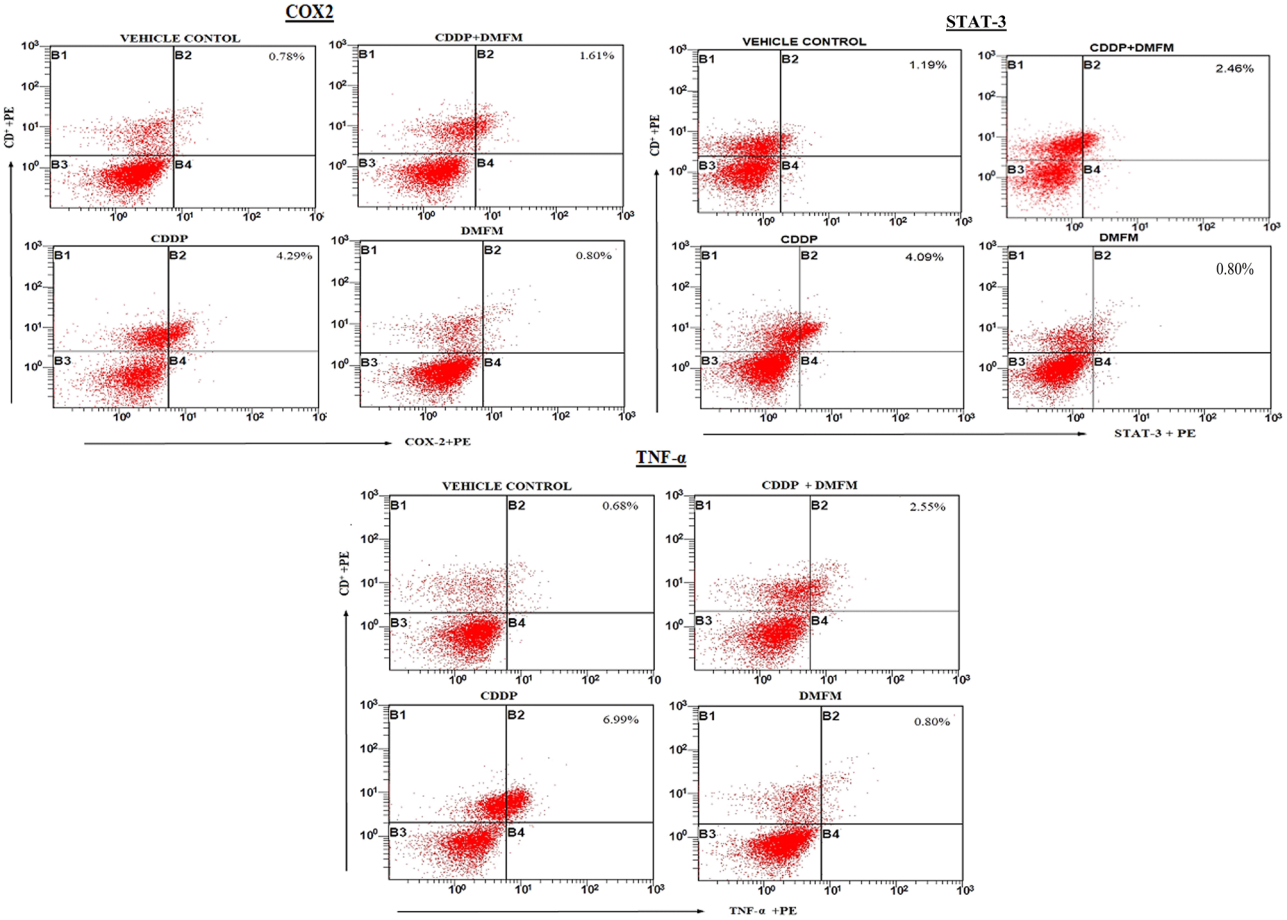
Influence of DMFM on CD4^+^COX2, CD4^+^STAT3, and CD4^+^TNF-α quantity. Flow cytometry study of CD4^+^COX2, CD4^+^STAT3, and CD4^+^TNF-α expression in whole blood. Representative dot plots for COX2, STAT3, TNF-α. and CD4^+^ expressing cells in whole blood from one rat from each group are presented.

DMFM mechanism of action was further evaluated by analysis of various T-cell populations using flow cytometry. There was a considerable increase in the proportion of double-positive CD4^+^STAT3, CD4^+^TNF-α, and CD4^+^COX-2 cells (Fig. 3) in the CDDP group in contrast to the control vehicle group. Significantly, administration of DMFM following CDDP treatment caused a notable reduction in the percentage of these CD4^+^STAT3, CD4^+^TNF-α, and CD4^+^COX-2 cells. Our results suggest that an anti-inflammatory effect of DMFM in the CDDP exposed animals might be due to suppressed induction of COX-2, TNF-α, and STAT3.

### Effect of DMFM on mRNA expression of inflammatory factors and pathways

**Figure 4:**
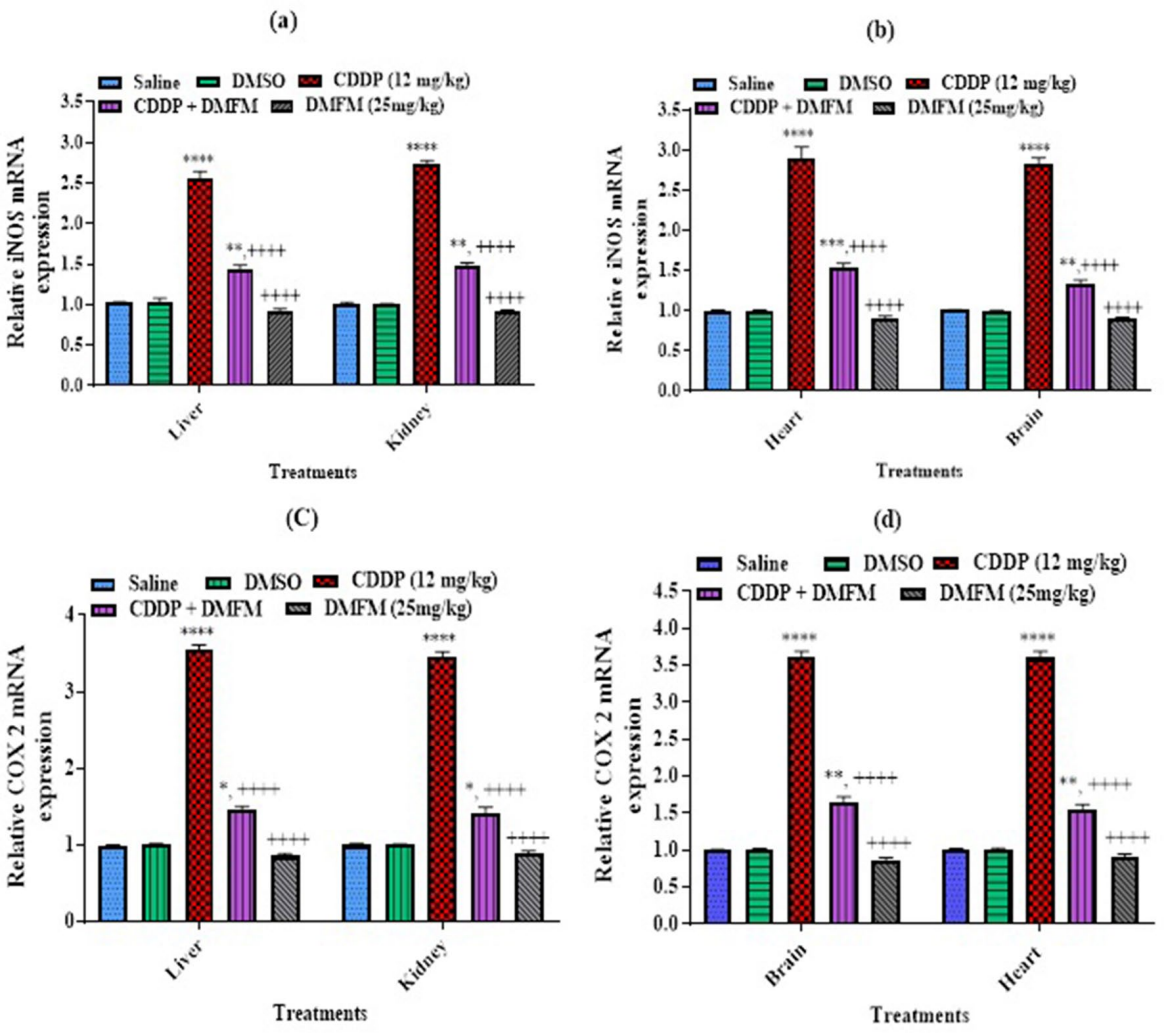
Quantification of mRNA expression of inflammatory mediators (COX-2, IL1 and iNOS) in various organs. *, **, ***, **** indicated *p* < 0.05, *p* < 0.01, *p* < 0.001 and *p* < 0.0001 versus saline group respectively, and ^++++^*P* < 0.0001 versus CDDP (12 mg/kg group). Statistics was carried out by one-way ANOVA followed by the Tukey- post-test. Each value indicates the mean ± SEM (n = 7).

**Figure 5:**
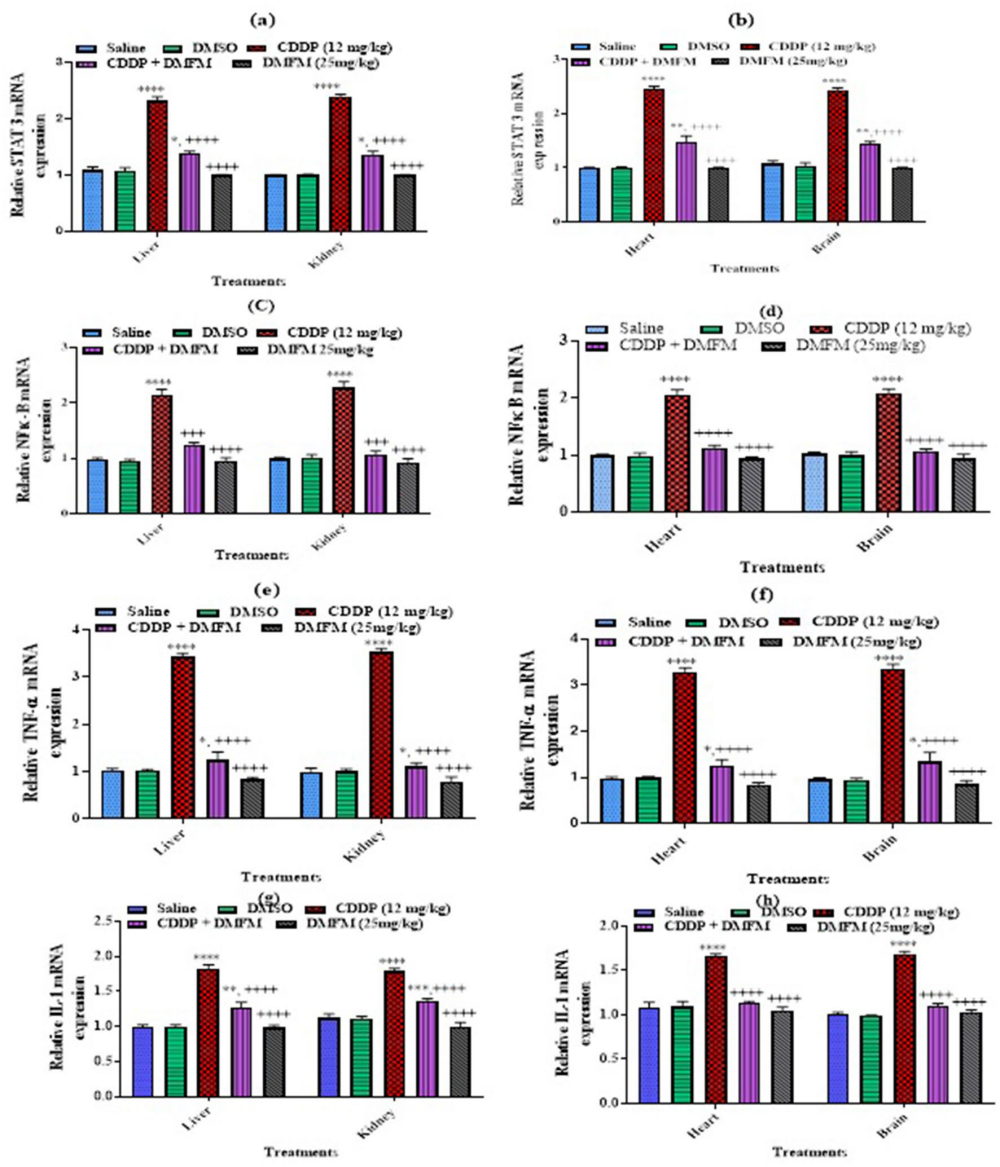
Quantification of mRNA expression of genes involved in the regulation of inflammatory markers. *, **, ***, **** indicated *p* < 0.05, *p* < 0.01, *p* < 0.001 and *p* < 0.0001 vs. saline group respectively, and ^++++^*P* < 0.0001 versus CDDP (12 mg/kg group). Statistics was carried out by one-way ANOVA followed by the Tukey- post-test. Each value indicates the mean ± SEM (n = 7).

We performed quantitative reverse transcriptase PCR (RT-PCR) analysis for inflammatory factors COX2 and iNOS gene expression levels. Our data demonstrated a prominent increase in the mRNA expression of these inflammatory markers in the liver, kidney, heart, and brain of CDDP-treated animals in contrast to the control group (Fig. 4). This CDDP-related elevation in mRNA expression was significantly attenuated by DMFM administration in all four tissues. COX2 mRNA is transcriptionally induced by IL 1 activation. NFκB and its upstream regulatory factor STAT3 initialize the signally cascade to activate COX2 and iNOS they are transcriptionally controlled by TNF alpha, NFκB, and STAT3. Therefore, we have assessed the expression of all the above factors. PCR analysis showed that CDDP considerably increased the mRNA expression of the inflammatory mediator’s genes in liver, kidney, heart, and brain tissues compared to control (Fig. 5). DMFM treatment significantly reduced CDDP-related expression of these regulatory genes in these tissues.

### DMFM regulates protein expression of inflammatory biomarkers

**Figure 6:**
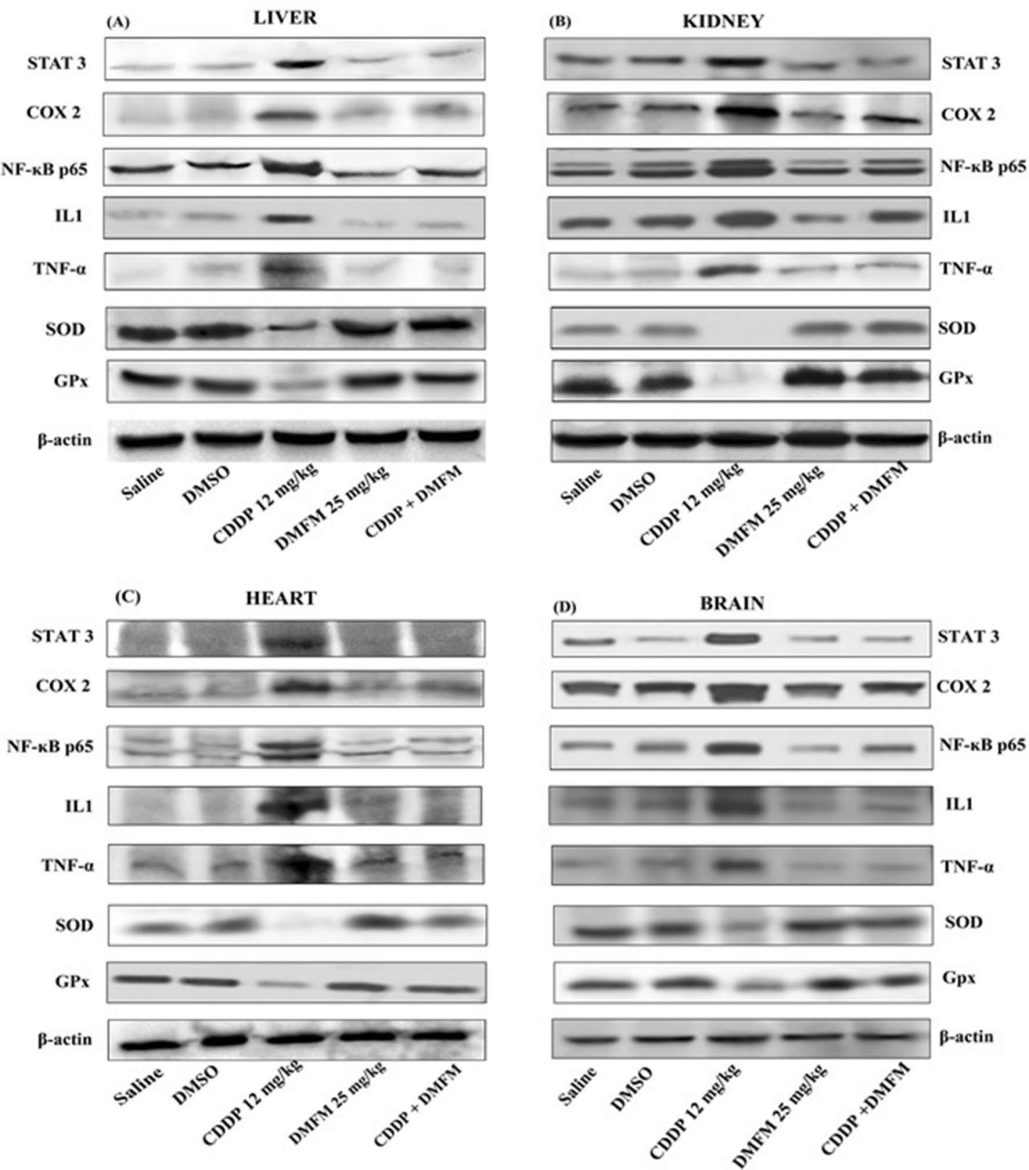
Protein immunoblotting analysis of tissue lysates prepared from (**A**) Kidney, (**B**) Liver, (**C**) Heart, and (**D**) Brain tissues treated with CDDP (12 mg/kg) and DMFM (25 mg/kg) or equal amount of DMSO as described in the experiment section. Groups are designated below the blots. Uncropped blots are included in Supplementary Fig. 1.

Changes in protein expression of inflammatory biomarkers were confirmed by immunoblot analysis. CDDP-induced inflammatory signaling changes were observed by marked elevation in brain, liver, kidney, and heart tissue protein levels for COX-2, TNF-α, NF-κB p65, STAT3, and IL-1 in contrast to control groups. As observed in the mRNA data, treatment of CDDP-exposed rats with DMFM considerably reversed the induction of STAT3, COX-2, TNF-α, NF-κB p65, and IL-1 proteins (Fig. 6).

### Alterations in protein expression of SOD and GPX by administration of DMFM

SOD and GPx protein expression levels were also evaluated in brain, liver, kidney, and heart tissues. In agreement with the activity data presented in Table 4, CDDP injection caused a dramatic loss of SOD and GPx protein levels relative to control, and treatment with DMFM following CDDP injection restored the expression of these proteins (Fig. 6).

### Histopathological examination of organs from the different treatment groups

**Figure 7:**
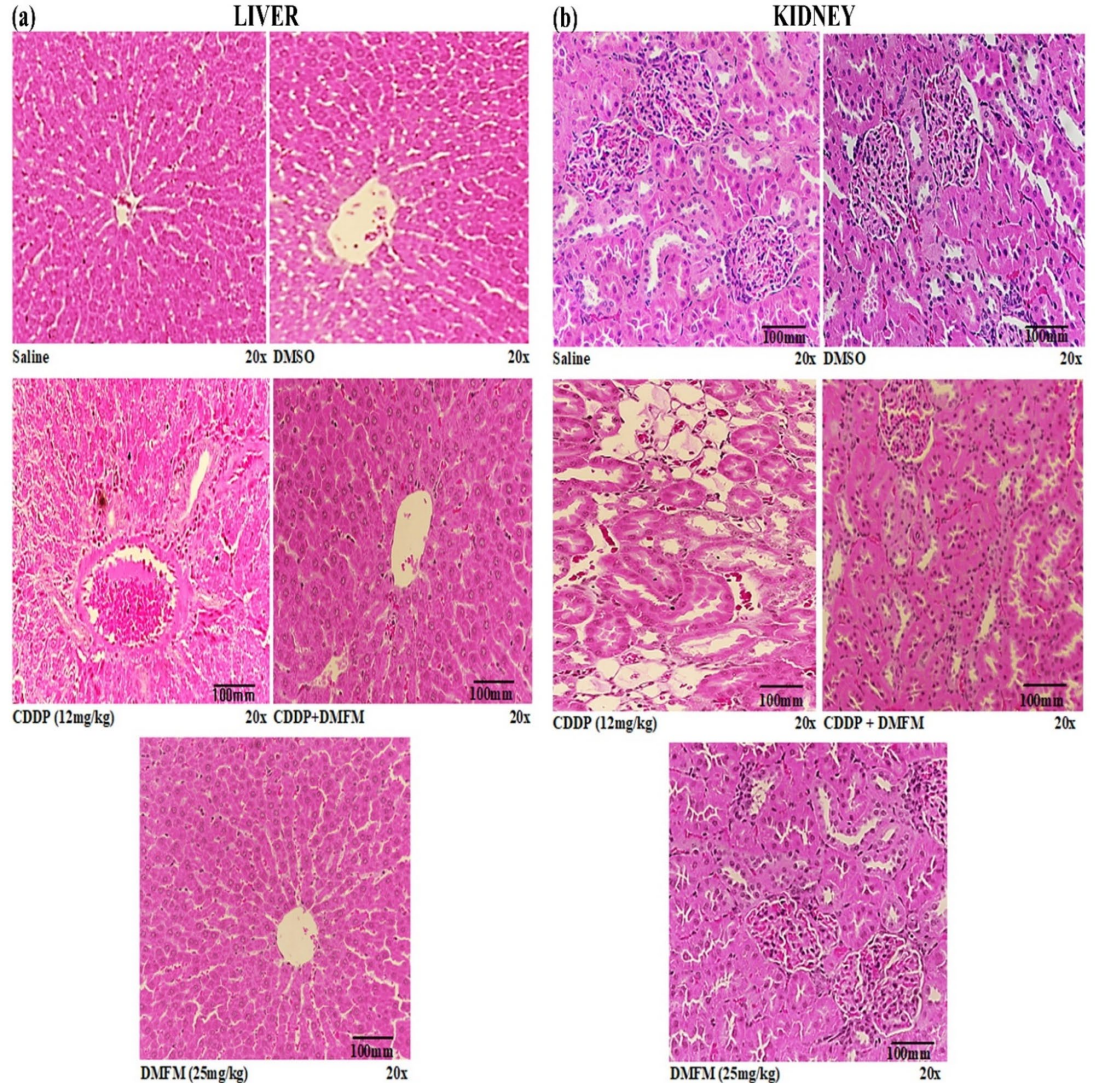
Representative H & E images of rat liver and kidney from all treatment groups. (**a**) Liver tissue sections from saline and DMSO groups present a typical hepatic structure. CDPP-treated liver sections revealed higher cellular lacerations, structural derangements, and assembly of inflammatory cells. DMFM + CDDP treated liver sections showed near to normal morphology. DMFM treatment showed normal morphology as in control rats. (**b**) Kidney section from saline and DMSO groups showing normal kidney morphology. CDPP-treated rat kidney section showing enhanced cellular lesions, ballooning, and collection of inflammatory cells. DMFM + CDDP treated sections with minor tubular deteriorations. DMFM alone treatment showed normal morphology as in control rats. Treatment conditions are indicated at the lower left of each image.

Histological examination of H & E stained hepatic tissue sections from CDDP-exposed animals demonstrated alterations in liver morphology i.e., leukocyte infiltration and cytoplasmic vacuolations. In comparison, rats that received DMFM after the CDDP dose presented more favorable liver histology with mild cytoplasmic vacuolations, leukocyte infiltration, and congestion (Fig. 7a).

Histological examination of H & E stained kidney sections indicated typical structures (glomeruli assembly encapsulated in Bowman’s capsule, tubules, interstitium, and blood vessels) in the control, DMSO and DMFM alone treated groups (Fig. 7b). The CDDP-treated group showed serious renal injuries, tubular necrosis, notable vacuolation, desquamation of epithelial cells in the renal tubules, enlargement of Bowman’s capsule, glomerular atrophy, pyknotic nuclei, proteinaceous casts in renal tubules, necrosis, detachment of the proximal tubular epithelial cell lining, detachment of the apical microvilli and improper cellular details. These impairments were considerably diminished by the use of post-CDDP DMFM treatment; specifically, kidneys from CDDP + DMFM treated rats possessed minor tubular deteriorations and the cellular arrangement seems typical with no proteinaceous cast. Thus, the CDDP-induced renal tubule system damage was largely ameliorated by DMFM.

**Figure 8:**
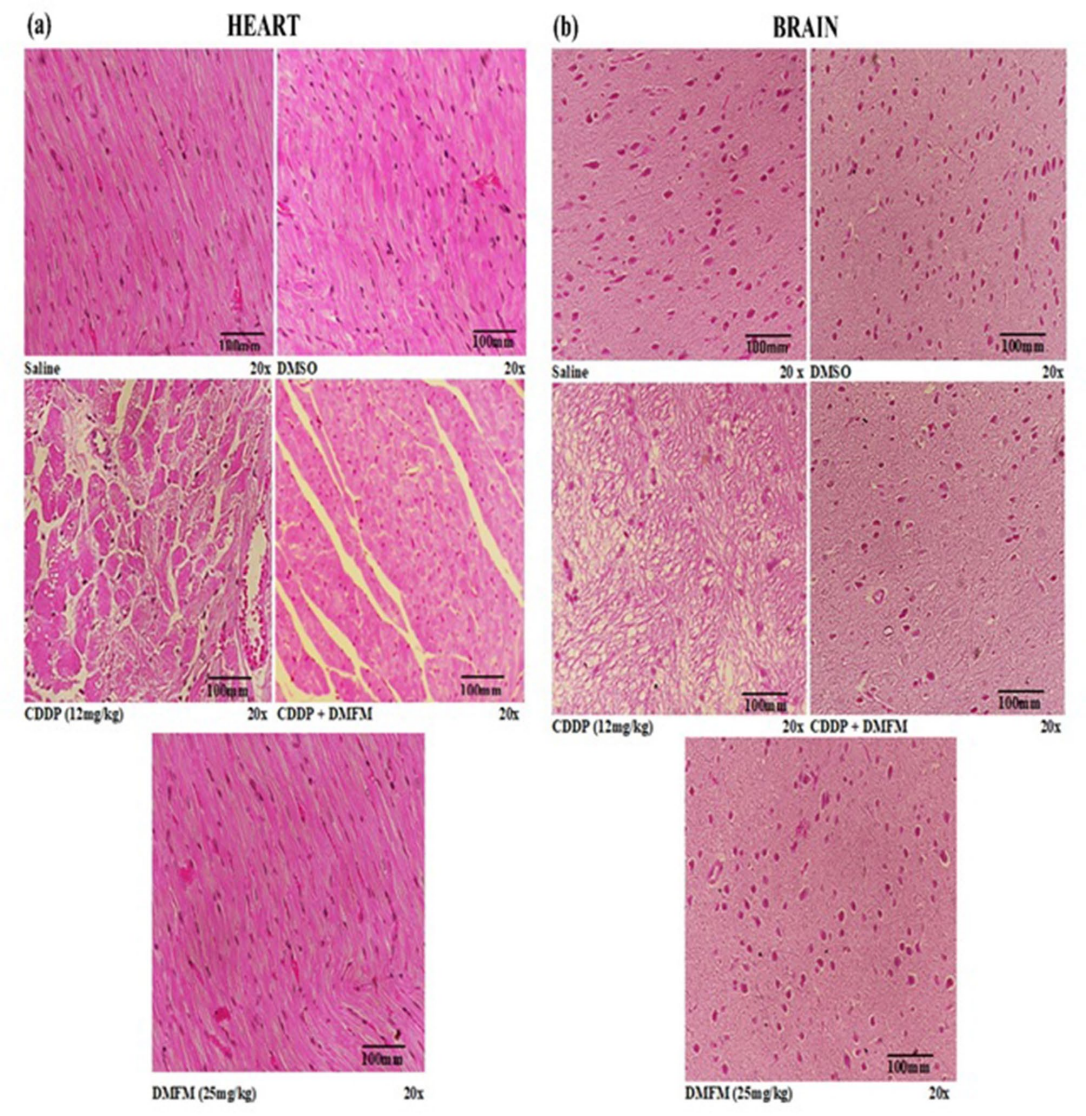
Representative H & E images of rat heart and brain from all treatment groups. (**a**) Cardiac muscle section from saline and DMSO groups displaying normal muscle fibers. CDPP-treated tissue sections depict greater cellular lesions and the presence of inflammatory cells. DMFM + CDDP treated heart induced significant injury. DMFM alone group exhibited regular morphology as in the saline group. (**b**) Brain sections from saline and DMSO groups presented typical morphology. CDPP-treated brain shows significant damage. DMFM treatment greatly improved brain tissue morphology. Treatment conditions are indicated at the lower left of each image.

Figure 8 depicts H & E images of heart and brain tissue sections from the various treatment groups. Cardiac sections of saline, DMSO, and DMFM treated groups exhibit the typical structure of cardiac muscle fibers with several delicate blood vessels, myofibrillar with striations, branched forms, assembly with neighboring myofibrils and capillaries in the connective tissue, and stable acidophilic cytoplasm with a central nucleus (Fig. 8a). CDDP treatment-induced enormous degenerative changes, including hypertrophy of muscle fibers, disruption in the trabeculae of the heart, and deteriorating lacerations in muscle fibers. Additionally, leukocyte infiltrations and vacuolated muscle fibers are observed. Heart from CDDP + DMFM treatment shows minor deterioration, with less capillary, vacuolar variations in contrast to the CDDP-alone group, and maximum muscle fiber presence compared to the saline group.

In brain tissue, the cerebellar cortex of saline, DMSO, and DMFM alone groups displayed typical histopathology with deeply basophilic Purkinje cells. CDDP injection triggered serious multifocal histological irregularities in the cerebral cortex. The Purkinje cells of the CDDP group were contracted with roughly presenting karyolysis and peri-cellular halos in contrast to the saline group. Numerous vacuoles of various sizes were seen amid most of the cells in all layers. Co-treatment with DMFM greatly improved brain tissue morphology with the majority of cells similar in appearance to the saline group. Small numbers of shrunken, darkly stained nuclei, pericellular halos, and vacuolated neuropil were observed in the CDDP + DMFM tissue.

### Three-dimensional structure of target molecules

**Figure 9:**
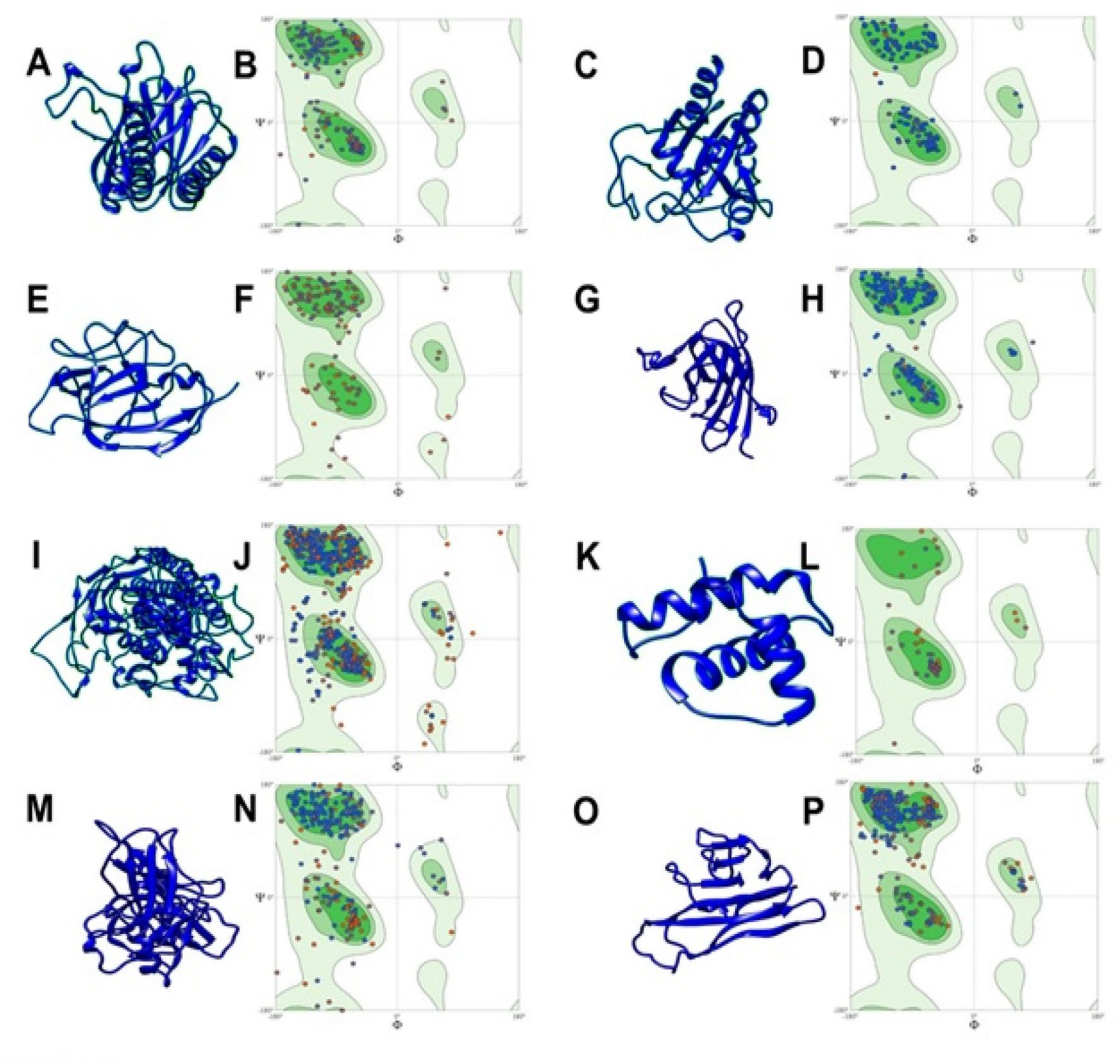
Modeling of three-dimensional structures for rat proteins. (**A**) 3D image of COX2. **(B**) Ramachandran plot of COX2. **(C)** 3D image of GPx. **(D)** Ramachandran plot of GPx. **(E)** 3D image of IL-1. (**F**) Ramachandran plot of IL-1. **(G)** 3D image of SOD. **(H)** Ramachandran plot of SOD. (**I**) 3D image of STAT3. **(J)** Ramachandran plot of STAT3. **(K)** 3D image of NF-κB. (**L**) Ramachandran plot of NF-κB. **(M)** 3D image of P65. **(N**) Ramachandran plot of P65. (**O**) 3D image of TNF-α. **(P)** Ramachandran plot of TNF-α.

Due to the unavailability of *Rattus norvegicus* crystal structures for NF-κB, TNF-α, P65 GPX, SOD, IL1, STAT3, and COX2, the I-Tasser server was used to predict three-dimensional models for further computational studies. The results obtained indicated good topology with a minimal number of outliers, hence they were predicted as good quality models (Fig. 9).

### Molecular docking interactions

The docking interaction of DMFM with **COX2** revealed strong interactions with a binding energy of − 42.00 kcal/mol (Table 5). ALA-182, THR-183, and GLU-198 formed a covalent bond with dimethylaminobenzylidene rings. Apart from these, GLY-169, LYS-171, and ASP-173 residues were involved in several van der Waals interactions as reported in Table 5.

**Table 5:**
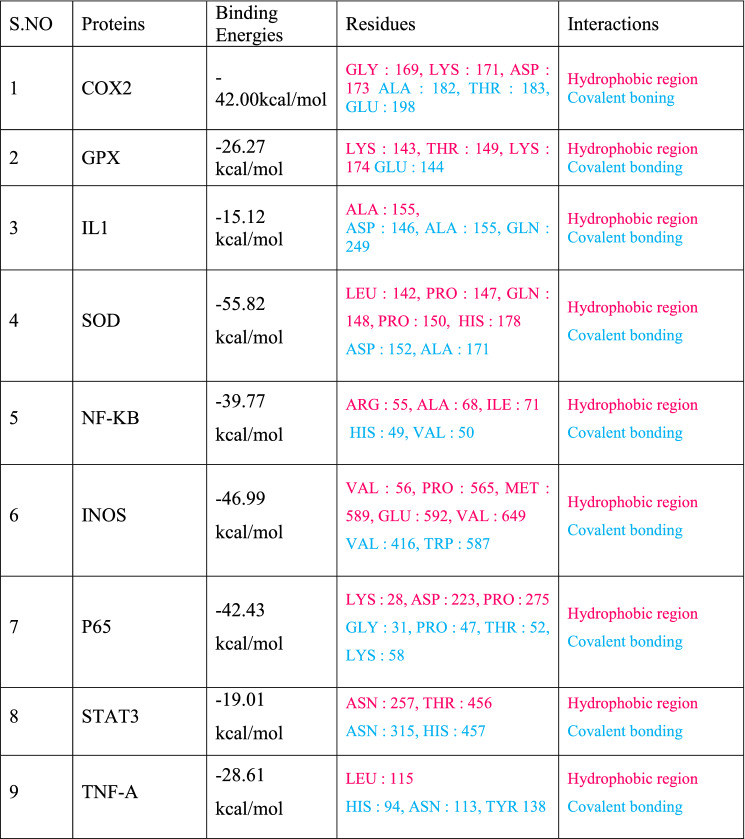
Binding residues and types of interactions of DMFM with all Target proteins.

Blue font colour shows covalent bonding and pink colour shows the residues involved in hydrophobic bonding.

**Table 6:** Molecular descriptors of DMFM through *insilico* QSAR model.

Drug	Molecular weight	Num.heavy atoms	Fraction csp3	Num.rotatible bonds	Num. H bonds acceptor	Num. H bond donor	Molar refractivity	TPSA	Formula
Physiochemical properties	501.61 g/mol	36	0.17	8	3	3	146.76	80.98	C29H28FN3O2S
Pharmacokinetics	GI absorption	BBB permeant	P-gp substrate	CYP1A2 inhibitor	CYP2C19 inhibitor	CYP2C9 inhibitor	CYP2D6 inhibitor	CYP3A4 inhibitor	Log Kp (skin permeation)
	Low	No	No	No	Yes	Yes	No	Yes	− 6.54 cm/s
Water solubility	Log S (ESOL)	Solubility	Class	Log S (SILICOS-IT)	Solubility	Class			
	− 5.29	2.55e−03 mg/ml ; 5.09e−06 ml/l	Moderately soluble	− 9.76	8.68e−08 mg/ml; 1.73e-10 ml/l	Poorly soluble			
Druglikeness	Lipinski	Ghose	Veber	Egan	Muegge	Bioavailibility score			
	No, 2 violations: MW > 500, MLOGP > 4.15	No, 3 violations: MW > 480, WLOGP > 5.6, MR > 130	Yes	No, 1 violation: WLOGP > 5.88	Yes	0.17			

**Figure 10:**
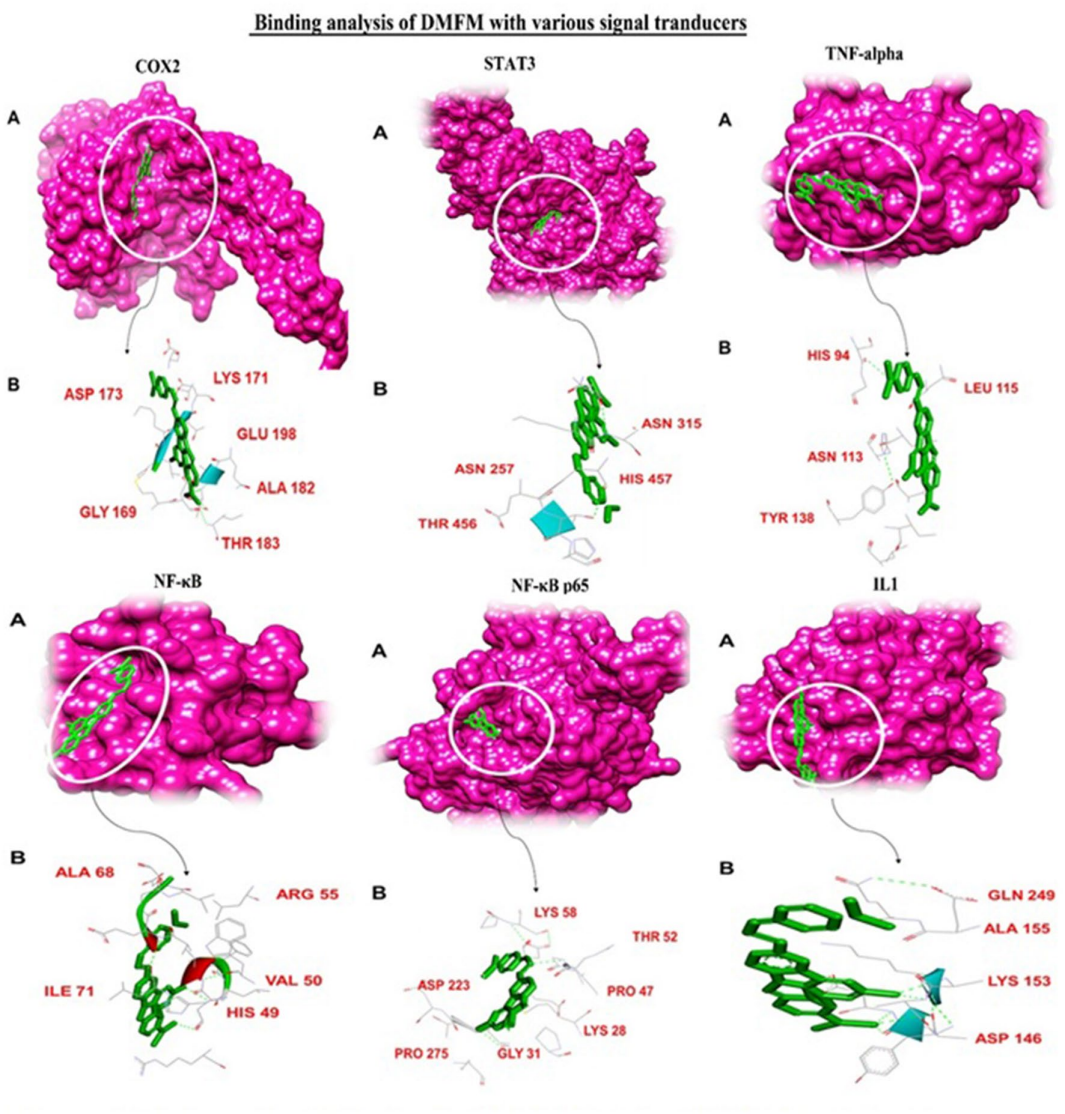
Binding analysis of DMFM with COX2, STAT3, TNF-α, NF-κB, NF-κB p65, and IL-1: (**A**) For each protein, a hydrophobic model is shown in purple color in 3D conformation while the docked DMFM compound is shown in green as a stick model. **(B)** In the 2D image, the DMFM compound is shown as a green stick model, and interacting residues of the protein are shown as grey sticks with H-bonding depicted as green dotted lines. Protein labels are indicated above each set of panels.

Molecular docking of DMFM with **STAT3** showed a strong affinity with a binding energy of − 19.01 kcal/mol. ASN-315 and HIS-457 showed conventional hydrogen bonding with dimethyl aminobenzylidene and acetohydrazide moieties of the DMFM compound together with hydrophobic interactions by ASN-257 and THR-456 residues (Fig. 10; Table 5).

Detailed docking interaction analysis of DMFM within the **TNF-α** binding cavity revealed a high affinity with a binding energy of − 28.61 kcal/mol. HIS-94, ASN-113 and TYR-138 residues of TNF-α made hydrogen bonds with the acetohydrazide and dimethylaminobenzylidene moieties of the DMFM compound. LEU-115 of TNF-α contributed to hydrophobic interactions to keep the drug within the binding cavity (Fig. 10).

DMFM also showed a good binding affinity with the **NF-κB** transcription factor. HIS-49, VAL-50, ARG-55, ALA-68, and ILE-71 of NF-κB showed covalent and hydrophobic interactions with a binding energy of − 39.77 kcal/mol (Fig. 10; Table 5).

Docking analysis of DMFM showed a high affinity towards **the P65** binding cavity with a binding energy of − 42.43 kcal/mol (Table 5). GLY-31 formed H-bonds with dimethylamino benzylidene, PRO-47, THR-52, and LYS-58 with fluoro moiety of DMFM compound. LYS-28, ASP-223, and PRO-275 of the P65 binding cavity showed van der Waals interactions with DMFM (Fig. 10).

DMFM compound interaction with IL-1 showed covalent and van der Waals interactions having a binding energy of − 15.12 kcal/mol (Table 5). ASP-146, ALA-155, and GLN-249 formed H-bonds with the acetohydrazide and dimethylaminobenzylidene moiety of the DMFM compound. The ALA-155 residue was involved in hydrophobic interaction (Fig. 10).

**Figure 11:**
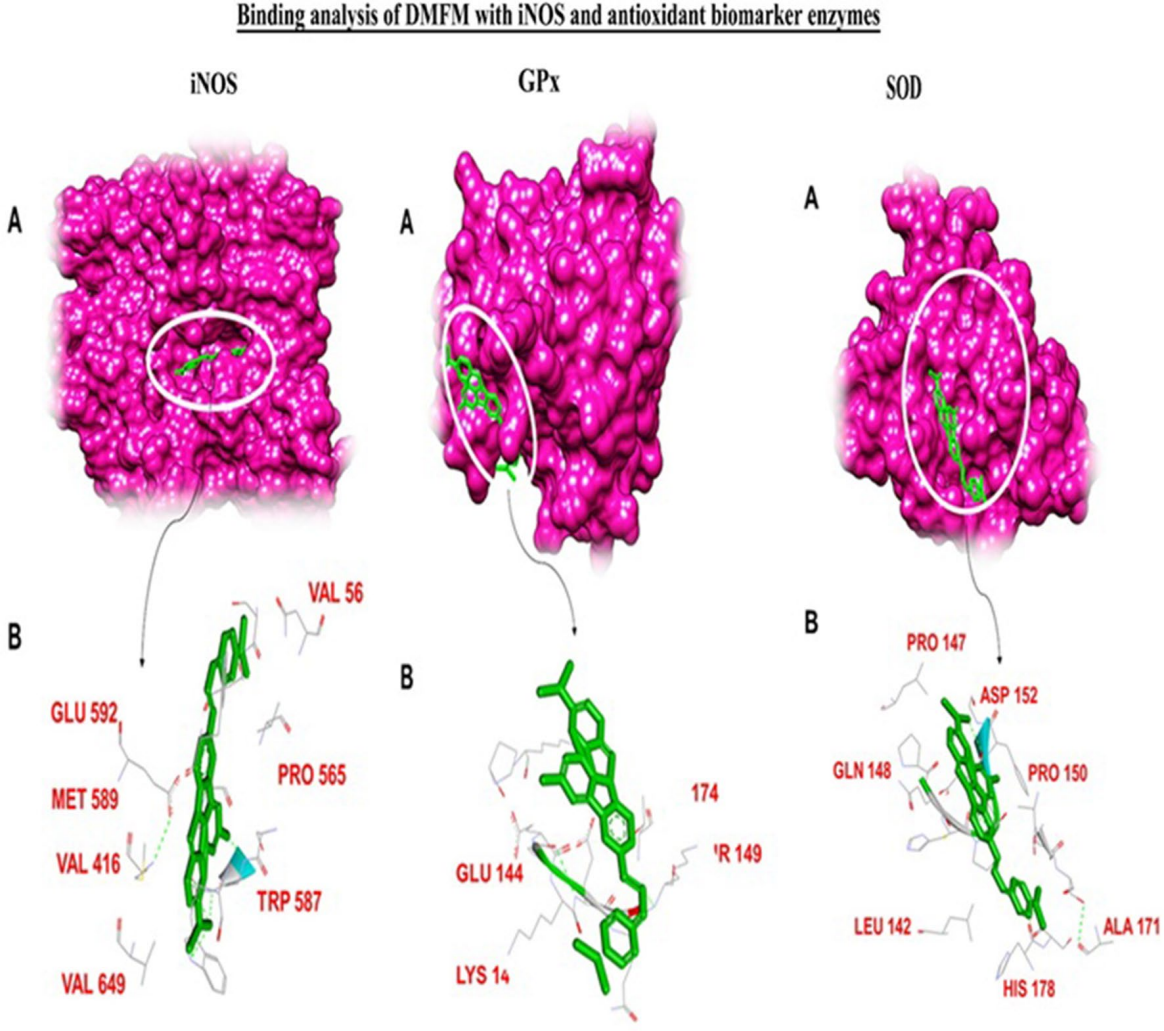
Binding analysis of DMFM with iNOS, GPx, and SOD protein. (**A**) In the 3D conformation, protein is shown in purple font while the DMFM is shown in the green model. (**B**) In 2D representation green stick model represent the DMFM, all the binding residues are shown in the gray line model while the green dotted lines represent H-bonding.

DMFM drug showed a high binding affinity towards **iNOS** binding cavity with binding energy 46.99 kcal/mol (Table 5). VAL-416 and TRP-587 build hydrogen bonds with dimethylaminobenzylidene and methyl ring of DMFM. Apart from these, VAL-56, PRO-565, MET-589, GLU-592, and VAL-649 showed hydrophobic interactions with the drug molecule (Fig. 11).

Binding interactions of DMFM with **GPx** showed high affinity towards the binding cavity of GPx (LYS-143, THR-149, LYS-174, GLU-144) having a binding energy of − 26.27 kcal/mol (Table 5). GLU-144 of GPx formed H-bond with the acetohydrazide moiety of DMFM. LYS-143, THR-149, and LYS-174 of the GPx protein made hydrophobic interactions with the DMFM molecule to hold it within the cavity (Fig. 11).

DMFM was bound to **SOD** with a high-affinity binding energy of − 55.82 kcal/mol (Table 5). ASP-152 and ALA-171 made conventional hydrogen bonds with acetohydrazide and dimethyl aminobenzylidene of the DMFM compound. LEU-142, PRO-147, GLN-148, PRO-150, and HIS-178 residues showed van der Waals interactions with the docked inhibitor (Fig. 11).

### QSAR model

**Figure 12:**
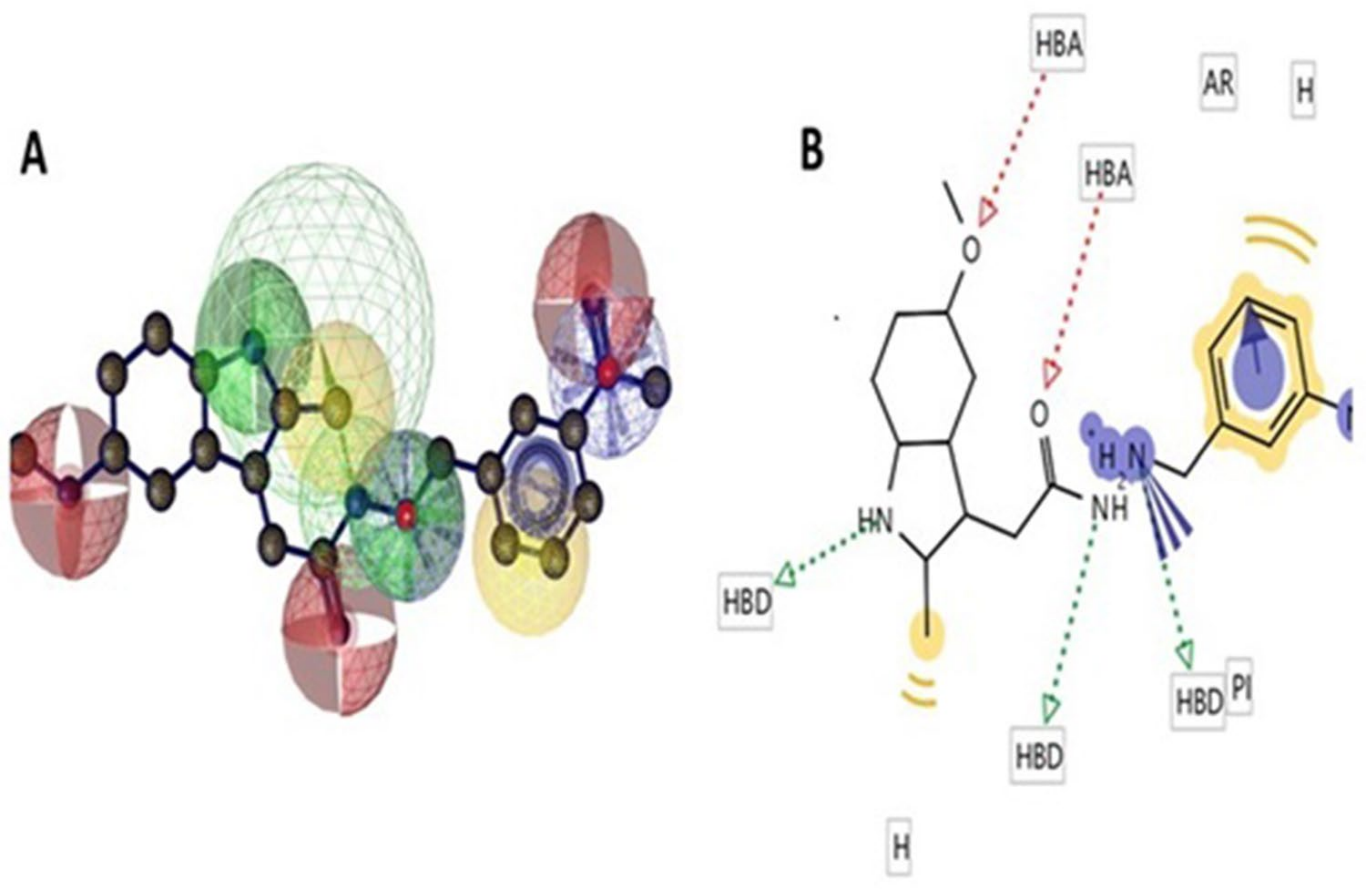
Pharmacophore model of DMFM compound. (**A**) 3D representation of DMFM pharmacophore model. Red spheres represent hydrogen bond acceptor (HBA), green spheres represent hydrogen bond donor (HBD), and yellow spheres depict aromatic rings. (**B**) The same color theme with the same descriptors is represented in 2D conformation.

A QSAR (quantitative structure–activity relationship) model of DMFM was computed to gain knowledge of its structural properties to assess potential biological impacts. Using these computational tools and servers the molecular descriptors of DMFM were measured (Fig. 12 and Table 6). The DMFM drug showed a molecular weight of 501.61 g/mol, with 36 heavy atoms. Three hydrogen bond donors and three hydrogen bond acceptors were measured, as well as 8 rotatable bonds. The negative and positive ionizable elements were 0 and 2, respectively. The cLog *P* value of DMFM was estimated as 3.021.

### Molecular dynamic simulation

**Figure 13:**
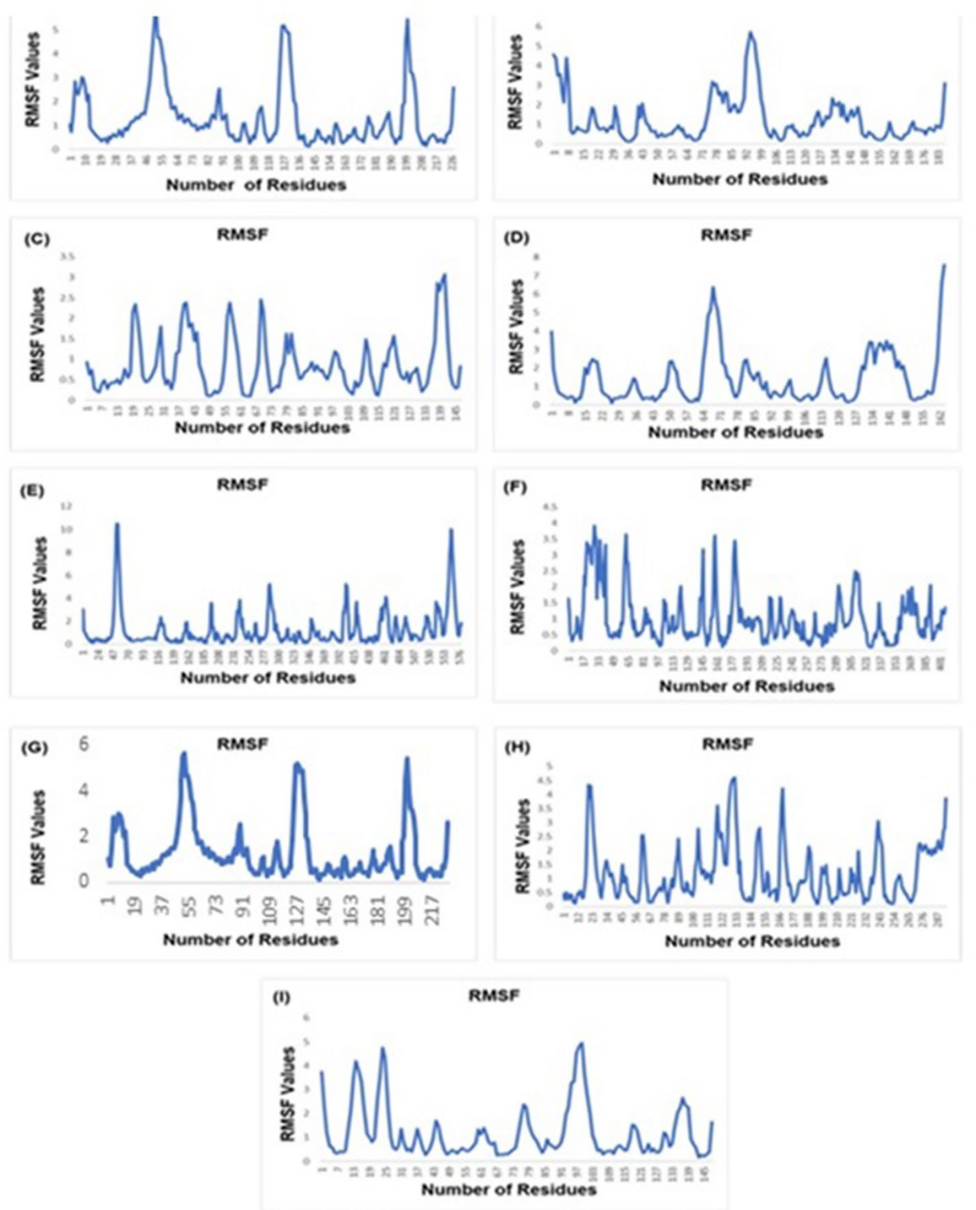
The RMSF plot of all the system trajectories. (**A**) COX2 (**B**) GPx, (**C**) IL-1 (**D**) SOD, (**E**) STAT3, (**F**) iNOS, (**G**) NF-κB, (**H**) p65 and (**I**) TNF-α.

All the docked systems were subjected to molecular dynamics simulations to investigate the stability of the interaction between drug and drug targets. The root means square fluctuation (RMSF) of COX2, IL-1, SOD, GPx, NF-κB, P65, STAT3, iNOS, and TNF-α systems were calculated to measure the fluctuations of all the protein C-alpha atoms. All the systems showed stability throughout the simulations in the dynamic trajectories generated at 30 ns (Fig. 13).

COX2 dynamic trajectories showed stability in the region comprising binding residues. Fluctuations were observed at regions ranging from 37–64, 113–136, and 190–208 with an RMSF value of 5.6 (Fig. 13A). Dynamic simulation analysis of GPx revealed fluctuation at diverse regions of the protein ranging from residues 1–8 and 92–106 with RMSF value 6. Interestingly, all the residues critical for drug binding showed stability in their behavior (Fig. 13B). Analysis of the IL-1 system showed fluctuation in the region of amino acids 133–145 of the protein. Remarkably, all fluctuations were observed in the region close to the binding cavity to aid in strong binding with favorable poses (Fig. 13C). The simulations trajectories of the SOD protein revealed fluctuations in the regions of residues 64–76 and 155–162 with a high RMSF value of 8 Å for the region encompassing amino acids 155–162 (Fig. 13D). STAT3 system trajectory showed fluctuations in the region encompassing 47–70 and 553–576 residues, while the important residues of the binding cavity showed stability (Fig. 13E). The iNOS dynamic trajectory showed less stability in the regions including 17–33, 145–161, and 177–193 (Fig. 13F). The dynamic trajectory of NF-κB showed fluctuation in the region ranging from residues 49–55 with a high RMSF of 4.5 Å (Fig. 13G). P65 protein trajectories showed fluctuation residues ranging from 12–23, 122–133, and 166–177 with an RMSF value of 4.5 Å (Fig. 13H). The dynamic trajectory of the TNF-α system revealed fluctuation in the protein regions 19–25 and 91–103 with an RMSF value of 5 Å (Fig. 13I). Overall, the binding region and critical binding residues of COX2, IL-1, SOD, GPx, NF-κB, P65, STAT3, iNOS, and TNF-α systems showed stability throughout the 30 ns simulations experiments. Most fluctuations observed around the binding site were favorable to binding poses.

## Discussion

In the current investigation, we have studied the damaging effect of CDDP on various organs and the protection provided by DMFM co-treatment. The most prevalent adverse effect associated with CDDP treatment is its nephrotoxicity. A single inoculation of CDDP could lead to a significant accumulation of the drug in the kidney for 1 month or even 3 months^53^. CDDP-induced nephrotoxicity is linked with morphological alterations, reduction in glomerular filtration rate, and increased creatinine and urea nitrogen (BUN) blood levels^54^. Hence we have examined biochemical markers of kidney damage besides histopathological evaluation. DMFM + CDDP treatment significantly ameliorated kidney function biomarkers in comparison to CDDP alone group. These functional deformities in CDDP exposed rats are indicators of kidney tubular cell malfunctioning or lipid peroxidation and free radical production in nephrons^55–58^. Therefore, we studied oxidant/antioxidant biomarkers in various tissues of treatment groups. We have recorded redox imbalance in tissues of CDDP exposed animals. CDDP induced redox inconsistency, as corroborated by a rise in MDA and NO activity and reduced SOD and Gpx intensity in the renal, hepatic, brain, and cardiac tissue. CDDP accumulates in the mitochondria of kidney epithelial cells and induces ROS synthesis and decreases antioxidant enzymes in various tissues. It is still poorly defined How ROS has generated inside the cells after CDDP exposure and the contribution of ROS generation in CDDP induced cytotoxicity in normal versus cancer cells. However various mechanisms are probable, including the electron transport chain scheme in mitochondria, cytochrome P450 enzymes, and NADPH oxidase^59^.

This ROS generation after CDDP exposure severely affects the antioxidant levels i.e., GST, GSH-Px, GR, and SOD in the tissues. CDDP-induced toxicity has been related to the reduction of glutathione (GSH) which potentiates the CDDP-induced cytotoxicity^60^. Therefore, besides enzymatic quantitative analysis, the effect of various treatments on antioxidant biomarkers was validated by protein expression analysis as well. Previous research indicated that CDDP increases mitochondrial ROS generation by decreasing mitochondrial GSH and other antioxidant enzymes. Similarly, we have observed a significant decrease in protein expression of SOD and GPx in tissues of CDDP (12 mg/kg) exposed animals. Restoration of GPx and SOD expression levels in the DMFM + CDDP group indicated that DMFM prevented the oxidative injury induced by CDDP exposure by triggering antioxidant gene expression. Elevated levels of GPx and SOD in the CDDP + DMFM administration group might scavenge the free radicals which further shields the free radicals induced injury in various tissues. As various previous investigations approves that compounds with antioxidant activity have a protective effect against CDDP induced toxicity^61^. Comparable results for antioxidant enzymes (CAT, GPx, and SOD) and GSH expression in CDDP induced nephrotoxic rats have been reported^62,63^. Adachi and colleagues observed transitory intensification in scavenger activity, such as catalase, GPx, and SOD after Sulindac compound administration depending on the cell line^64^. The hydrogen and/or electron and proton transfer mechanism by the indole ring is possibly responsible for quenching free radicals and the antioxidant activity of DMFM. Previous studies also approved that radical stabilization is strongly reliant on the form of substituents on the indole moiety and their relative positions. In addition, the radical dissipation inside the indole system is mandatory for the observed antioxidant activity^65^. Aspartate transaminase (AST), alkaline phosphatase (ALP), and alanine aminotransferase (ALT) are extensively distributed all over the body, especially in the liver, where they act as sensitive indicators of hepatocellular degeneration, necrotic changes, and liver function^66^. In this study, CDDP hepatotoxicity was demonstrated by increased AST, ALP, and ALT along with histopathological changes, including inflammatory cell infiltrations, vacuolations, and degenerative changes. Similar to oxidative stress, inflammation is generally involved in the whole spectrum of liver diseases from initial to advanced stages. Our data are consistent with numerous earlier observations that demonstrated the hepatotoxic effect of CDDP and its associations with the increased free radical formation and subsequent oxidative stress^67^. Various studies have shown that Sulindac derivatives have hepatotoxic potential and the mechanism of action might be inflammatory stress-induced sensitivity to drug-induced liver injury^68^. The hepatic injury induced by Sulindac derivatives might be due to the upregulation of TNF-α and I-L1 mediated inflammatory responses. In contrast, we observed novel Sulindac derivative DMFM remarkably protects from hepatic injury via lowering serum ALT, AST, and ALP when combined with CDDP. One mechanism of action of DMFM might be its capability to act as a free radical scavenger in case of CDDP toxicities, thereby protecting membrane permeability. DMFM also generated possible hepatoprotective actions by decreasing inflammatory mediators (TNF-α, IL-1, and COX-2), increasing the hepatic enzyme activities of SOD and GPX, as well as down-regulating MDA levels. Also, our results proposed that DMFM reduces CDDP’s hepatotoxic effect by increasing antioxidant mechanisms.

COX2 prominently functions in pain and inflammation through prostaglandin formation, which in turn enhances inflammation via vasodilation and recruitment of inflammatory cells^69,70^. Therefore, we analyzed the DMFM effect on T-cell populations by flow cytometry. NF-κB controls the transcription of COX2 and iNOS which act as inflammatory mediators^71^. It was observed that Sulindac metabolites inhibit NF-kB-mediated signals and have ROS mediating potential without inhibiting COX2 in cancer cell lines^72^, however, we observed inhibition of NF-kB and COX2 in various tissues in vivo along with increased expression of antioxidant genes. Furthermore, DMFM suppressed the activation of NFκB and its upstream molecular factor, STAT 3. These results suggest that the protection mechanism of DMFM is by inhibition of NFκB activation possibly via STAT3. STAT3 is prominently associated with NF-κB signaling and these two molecules control numerous inflammatory genes and oncogenes^14,73^. NF-κB initiation has an essential role in the assembly of IL-1β^74–76^. Blocking NF-κB function leads to reduced expression of iNOS and subsequently might show encouraging therapeutic results for the behavior of inflammatory diseases. This supports our findings which demonstrate that DMFM suppresses iNOS and COX2 possibly via restraint of NFκB activation as seen in the immunoblot expression. RT-PCR analysis also demonstrated the downregulation of COX2, NF-κB, and iNOS in the CDDP + DMFM group in contrast to the CDDP group. Moreover, our results illustrated that CDDP treatment considerably increased the expression of IL-1 and TNF-α in the CDDP group as compared to the control. DMFM administration simultaneously with CDDP prominently reduced the expression of IL-1 and TNF-α, in the brain, heart, kidney, and liver tissue of the CDDP + DMFM group in contrast to the CDDP group, reflecting the anti-inflammatory efficacy of DMFM. This effect may cause a decline in organ toxicities and it could be a possible therapeutic against CDDP-induced organ toxicities^77^.

Our data demonstrated extreme neurotoxicity induced by CDDP in the rat brain which was confirmed by the increased activities of AChE, altered antioxidative/oxidative status, and increased secretion of pro-inflammatory cytokines^78–80^. The anti-inflammatory effect of DMFM by counterbalancing NF-κB and the antioxidant activity shown by DMFM by improving GSH may be crucial mechanisms behind the specified development in the pro-inflammatory cytokines.

CDDP-induced cardiotoxicity results from the disposition of CDDP in the sinoatrial node area that causes cardiomyocyte contraction, dysfunctioning of the left ventricle, and bradycardia,^81^. These anomalies can derive from inflammation, oxidative stress, ER stress, and apoptosis. Our data revealed that DMFM considerably reduces CDDP-mediated cardiac damage, as observed from the biochemical analysis of several cardiac markers like CKMB, and severe histopathological alterations. DMFM reduced ROS development in the heart of rats exposed to CDDP.

Finally, in silico molecular docking and dynamics simulations revealed high binding affinity and stability of DMFM towards GPx, COX2, IL-1, and NF-κB. The QSAR model of DMFM predicted it as a good drug with an inhibitory effect on the target molecules evaluated here.

## Conclusion

The present study aimed to examine the protective effects of DMFM against CDDP-induced organ toxicities as well as the therapeutic characteristics of the sulindac acetohydrazide, N'-(4-dimethylaminobenzylidene)-2-1-(4-(methylsulfinyl) benzylidene)-5-fluoro-2-methyl-1H-inden-3-yl) acetohydrazide (DMFM) during CDDP therapy. DMFM may counterbalance CDDP-induced nephrotoxicity, cardiotoxicity, nephrotoxicity, and neurotoxicity effects generally through anti-inflammatory and antioxidant mechanisms. In this pathway, the CDDP inoculation is chiefly rooted in the modulation of STAT3, IL-1, NF-κB, TNF-α, COX2, and iNOS transcription factors which are crucial watchdogs of inflammation and oxidative stress. Our results suggested that STAT3-NF кB-TNF-α and IL1 induced COX-2/iNOS_2_ upregulation is normalized by DMFM treatment. DMFM increase the mRNA and protein expression of antioxidant (SOD and Gpx). Hence DMFM via antioxidant defense mechanism protected CDDP induced inflammatory and toxic responses in various organs. We recommend that more in-depth studies are needed to more fully understand the molecular mechanisms of the protective effects of DMFM. These encouraging properties necessitate proof-of-concept clinical trials and additional efforts to study the protective effects of DMFM.

## Supplementary Information

Supplementary Information.

## Data Availability

All the data is contained in the manuscript.

## References

[1] Perše, M. & Večerić-Haler, Ž. J. B. R. I. Cisplatin-induced rodent model of kidney injury: Characteristics and challenges. BioMed Res. Int. **2018** (2018). 10.1155/2018/1462802 PMC6157122 30276200

[2] Naqshbandi A, Khan W, Rizwan S, Khan F (2012). Studies on the protective effect of flaxseed oil on cisplatin-induced hepatotoxicity. Hum. Exp. Toxicol..

[3] Yao X, Panichpisal K, Kurtzman N, Nugent K (2007). Cisplatin nephrotoxicity: A review. Am. J. Med. Sci..

[4] Florea A-M, Büsselberg D (2011). Cisplatin as an anti-tumor drug: cellular mechanisms of activity, drug resistance and induced side effects. Cancers.

[5] Wang D, Lippard SJ (2005). Cellular processing of platinum anticancer drugs. Nat. Rev. Drug Discov..

[6] Yang Z (2006). Cisplatin preferentially binds mitochondrial DNA and voltage-dependent anion channel protein in the mitochondrial membrane of head and neck squamous cell carcinoma: Possible role in apoptosis. Clin. Cancer Res..

[7] Galluzzi L (2014). Systems biology of cisplatin resistance: Past, present and future. Cell Death.

[8] El-Awady E-SE, Moustafa YM, Abo-Elmatty DM, Radwan A (2011). Cisplatin-induced cardiotoxicity: Mechanisms and cardioprotective strategies. Eur. J. Pharmacol..

[9] Hussein A, Ahmed AA, Shouman SA, Sharawy S (2012). Ameliorating effect of DL-α-lipoic acid against cisplatin-induced nephrotoxicity and cardiotoxicity in experimental animals. Drug Discov. Ther..

[10] Sawyer DB (2002). Role of oxidative stress in myocardial hypertrophy and failure. J. Mol..

[11] Dugbartey GJ, Peppone LJ, de Graaf IA (2016). An integrative view of cisplatin-induced renal and cardiac toxicities: Molecular mechanisms, current treatment challenges and potential protective measures. Toxicology.

[12] Silva, L. B. *et al.* The role of TNF-α as a proinflammatory cytokine in pathological processes. *Open Dent. J.* **13**(1) (2019).

[13] Zhang JM, An J (2007). Cytokines, inflammation and pain. Int. Anesthesiol. Clin..

[14] Ogura H (2008). Interleukin-17 promotes autoimmunity by triggering a positive-feedback loop via interleukin-6 induction. Immunity.

[15] Taniguchi K, Karin M (2018). NF-κB, inflammation, immunity and cancer: Coming of age. J. Nat. Rev. Immunol..

[16] Chen L (2018). Inflammatory responses and inflammation-associated diseases in organs. Oncotarget.

[17] Ernst M (2008). STAT3 and STAT1 mediate IL-11–dependent and inflammation-associated gastric tumorigenesis in gp130 receptor mutant mice. J. Clin. Investig..

[18] Olcaydu D (2009). A common JAK2 haplotype confers susceptibility to myeloproliferative neoplasms. Nat. Genet..

[19] Lee H (2009). Persistently activated Stat3 maintains constitutive NF-κB activity in tumors. Cancer Cell.

[20] Yang J (2007). Unphosphorylated STAT3 accumulates in response to IL-6 and activates transcription by binding to NFκB. Genes.

[21] Khatri CK, Indalkar KS, Patil CR, Goyal SN, Chaturbhuj GUJB (2017). Novel 2-phenyl-4, 5, 6, 7-tetrahydro [b] benzothiophene analogues as selective COX-2 inhibitors: Design, synthesis, anti-inflammatory evaluation, and molecular docking studies. Med. Chem. Lett..

[22] Bindu S, Mazumder S, Bandyopadhyay U (2020). Non-steroidal anti-inflammatory drugs (NSAIDs) and organ damage: A current perspective. Biochem. Pharmacol.

[23] Bhat MA (2020). Novel sulindac derivatives: synthesis, characterisation, evaluation of antioxidant, analgesic, anti-inflammatory, ulcerogenic and COX-2 inhibition activity. J. Enzyme Inhib. Med. Chem..

[24] Romeiro NC (2009). Synthesis, pharmacological evaluation and docking studies of new sulindac analogues. Eur. J. Med. Chem..

[25] Fogli S (2010). Therapeutic potential of sulindac hydroxamic acid against human pancreatic and colonic cancer cells. Eur. J. Med. Chem..

[26] Mathew B, Hobrath JV, Connelly MC, Guy RK, Reynolds RC (2017). Diverse amide analogs of sulindac for cancer treatment and prevention. Bioorgan. Med. Chem. Lett..

[27] Mathew B, Snowden TS, Connelly MC, Guy RK, Reynolds RC (2018). A small diversity library of α-methyl amide analogs of sulindac for probing anticancer structure-activity relationships. Bioorgan. Med. Chem. Lett..

[28] Liedtke AJ (2012). Cyclooxygenase-1-selective inhibitors based on the (E)-2′-des-methyl-sulindac sulfide scaffold. J. Med. Chem..

[29] Felts AS (2008). Sulindac derivatives that activate the peroxisome proliferator-activated receptor γ but lack cyclooxygenase inhibition. J. Med. Chem..

[30] Bhat MA (2015). Design and synthesis of n-arylphthalimides as inhibitors of glucocorticoid-induced tnf receptor-related protein, proinflammatory mediators, and cytokines in carrageenan-induced lung inflammation. J. Med. Chem..

[31] Guideline, O. O. J. O. G. f. t. T. O. C. 425: Acute oral toxicity—up-and-down procedure. *OECD Guidelines for the Testing of Chemicals*, **2**, 12–16 (2001).

[32] Wilhelm, K.-P. & Maibach, H. I. J. D. OECD guidelines for testing of chemicals. In Dermatotoxicology, 509–511. CRC Press (2012).

[33] Bhat MA (2020). Novel sulindac derivatives: Synthesis, characterisation, evaluation of antioxidant, analgesic, anti-inflammatory, ulcerogenic and COX-2 inhibition activity. J. Enzyme Inhib. Med. Chem..

[34] Amin, A. *et al.* A standardized extract of Ginkgo biloba neutralizes cisplatin-mediated reproductive toxicity in rats. *J. Biomed. Biotechnol.* **2012** (2012). 10.1155/2012/362049 PMC3364708 22675250

[35] Molina, A. *et al.* Analyses of anaesthesia with ketamine combined with different sedatives in rats. *Vet. Med.* **60**(7) (2015).

[36] Afsar T, Razak S, Almajwal A (2019). Effect of Acacia hydaspica R. Parker extract on lipid peroxidation, antioxidant status, liver function test and histopathology in doxorubicin treated rats. Lipids Health Dis..

[37] Afsar T, Razak S, Almajwal A, Khan MR (2018). Acacia hydaspica R Parker ameliorates cisplatin induced oxidative stress, DNA damage and morphological alterations in rat pulmonary tissue. BMC Complement Altern Med..

[38] Razak S (2020). GCMS fingerprinting, in vitro pharmacological activities, and in vivo anti-inflammatory and hepatoprotective effect of selected edible herbs from Kashmir valley. J. King Saud Univ. Sci..

[39] Tsikas D (2017). Assessment of lipid peroxidation by measuring malondialdehyde (MDA) and relatives in biological samples: Analytical and biological challenges. Anal. Biochem..

[40] Ellman GL, Courtney KD, Andres V, Featherstone RM (1961). A new and rapid colorimetric determination of acetylcholinesterase activity. Biochem. Pharmacol..

[41] Kruger NJ (1994). Basic Protein and Peptide Protocols.

[42] Trembley JH (2011). Systemic administration of antisense oligonucleotides simultaneously targeting CK2α and α′ subunits reduces orthotopic xenograft prostate tumors in mice. Mol. Cell. Biochem..

[43] Razak S (2018). Taxifolin, a natural flavonoid interacts with cell cycle regulators causes cell cycle arrest and causes tumor regression by activating Wnt/β-catenin signaling pathway. BMC Cancer.

[44] Razak S, Afsar T, Almajwal A, Alam I, Jahan S (2019). Growth inhibition and apoptosis in colorectal cancer cells induced by Vitamin D-Nanoemulsion (NVD): Involvement of Wnt/β-catenin and other signal transduction pathways. Cell Biosci..

[45] Razak S (2021). Molecular docking, pharmacokinetic studies, and in vivo pharmacological study of indole derivative 2-(5-methoxy-2-methyl-1H-indole-3-yl)-N′-[(E)-(3-nitrophenyl) methylidene] acetohydrazide as a promising chemoprotective agent against cisplatin induced organ damage. Sci. Rep..

[46] Pettersen EF (2004). UCSF Chimera–a visualization system for exploratory research and analysis. J. Comput. Chem..

[47] Morris GM (2009). AutoDock4 and AutoDockTools4: Automated docking with selective receptor flexibility. J. Comput. Chem..

[48] Duan Y (2003). A point-charge force field for molecular mechanics simulations of proteins based on condensed-phase quantum mechanical calculations. J. Comput. Chem..

[49] Zlenko DJB (2012). Diffusion factor calculation for TIP4P model of water. Biofizika.

[50] Humphrey W, Dalke A, Schulten K (1996). VMD: visual molecular dynamics. J. Mol. Graph..

[51] Lipinski CA (2001). Avoiding investment in doomed drugs. Curr. Drug Discov..

[52] Shahid F (2017). Oral administration of Nigella sativa oil ameliorates the effect of cisplatin on brush border membrane enzymes, carbohydrate metabolism and antioxidant system in rat intestine. Exp. Toxicol. Pathol..

[53] Esteban-Fernández D, Verdaguer J, Ramirez-Camacho R, Palacios M, Gómez-Gómez MM (2008). Accumulation, fractionation, and analysis of platinum in toxicologically affected tissues after cisplatin, oxaliplatin, and carboplatin administration. J. Anal. Toxicol..

[54] Ugur S (2015). The renoprotective effect of curcumin in cisplatin-induced nephrotoxicity. Ren. Fail..

[55] Nazıroǧlu M, Karaoğlu A, Aksoy AO (2004). Selenium and high dose vitamin E administration protects cisplatin-induced oxidative damage to renal, liver and lens tissues in rats. Toxicology.

[56] Ateşşahín A, Çeríbaşi AO, Yuce A, Bulmus Ö, Çikim G (2007). Role of ellagic acid against cisplatin-induced nephrotoxicity and oxidative stress in rats. Basic Clin. Pharmacol. Toxicol..

[57] Naqshbandi A, Khan MW, Rizwan S, ur Rehman S, Khan F (2012). Studies on the protective effect of dietary fish oil on cisplatin induced nephrotoxicity in rats. Food Chem. Toxicol..

[58] Afsar T (2021). Acacia hydaspica R. Parker ethyl-acetate extract abrogates cisplatin-induced nephrotoxicity by targeting ROS and inflammatory cytokines. Sci. Rep..

[59] Zhang J, Ye Z-W, Tew KD, Townsend DM (2021). Cisplatin chemotherapy and renal function. Adv. Cancer Res..

[60] De Luca A (2019). A structure-based mechanism of cisplatin resistance mediated by glutathione transferase P1–1. PNAS.

[61] Nasr AY (2014). Protective effect of aged garlic extract against the oxidative stress induced by cisplatin on blood cells parameters and hepatic antioxidant enzymes in rats. Toxicol. Rep..

[62] El-Beshbishy HA, Bahashwan SA, Aly HA, Fakher HA (2011). Abrogation of cisplatin-induced nephrotoxicity in mice by alpha lipoic acid through ameliorating oxidative stress and enhancing gene expression of antioxidant enzymes. Eur. J. Pharmacol..

[63] An Y, Xin H, Yan W, Zhou X (2011). Amelioration of cisplatin-induced nephrotoxicity by pravastatin in mice. Exp. Toxicol. Pathol..

[64] Adachi, M. *et al.* Nonsteroidal anti-inflammatory drugs and oxidative stress in cancer cells. *Histol. Histopathol.* (2007). 10.14670/HH-22.437 17290354

[65] Afsar T, Razak S, Almajwal A, Khan MR (2018). Acacia hydaspica R. Parker ameliorates cisplatin induced oxidative stress, DNA damage and morphological alterations in rat pulmonary tissue. BMC Complement. Altern. Med..

[66] Gowda, S. *et al.* A review on laboratory liver function tests. *Pan Afr. Med. J.* **3** (2009). PMC2984286 21532726

[67] Afsar T, Razak S, Almajwal A, Khan MR (2018). Acacia hydaspica R Parker ameliorates cisplatin induced oxidative stress, DNA damage and morphological alterations in rat pulmonary tissue. BMC Complement. Altern Med..

[68] Whittaker S, Amar J, Wanless I, Heathcote J (1982). Sulindac hepatotoxicity. Gut.

[69] Tang C (2018). The roles of inflammatory mediators and immunocytes in tendinopathy. J. Orthop. Transl..

[70] Medzhitov R (2008). Origin and physiological roles of inflammation. Nature.

[71] Surh Y-J (2001). Molecular mechanisms underlying chemopreventive activities of anti-inflammatory phytochemicals: down-regulation of COX-2 and iNOS through suppression of NF-κB activation. Mutat. Res./Fundam. Mol. Mech. Mutagen..

[72] Yousef M, Saad A, El-Shennawy L (2009). Protective effect of grape seed proanthocyanidin extract against oxidative stress induced by cisplatin in rats. Food Chem. Toxicol..

[73] Yu H, Jove R (2004). The STATs of cancer—new molecular targets come of age. Nat. Rev. Cancer.

[74] Bollrath J (2009). gp130-mediated Stat3 activation in enterocytes regulates cell survival and cell-cycle progression during colitis-associated tumorigenesis. Cancer Cell.

[75] Grivennikov S (2009). IL-6 and Stat3 are required for survival of intestinal epithelial cells and development of colitis-associated cancer. Cancer Cell.

[76] Samavati L (2009). STAT3 tyrosine phosphorylation is critical for interleukin 1 beta and interleukin-6 production in response to lipopolysaccharide and live bacteria. Mol. Immunol..

[77] Jaggi AS, Singh N (2012). Mechanisms in cancer-chemotherapeutic drugs-induced peripheral neuropathy. Toxicology.

[78] Kadhim HJ, Duchateau J, Sébire G (2008). Cytokines and brain injury: Invited review. J. Intensive Care Med..

[79] Chen C (2019). Ginsenoside Rb1 ameliorates cisplatin-induced learning and memory impairments. J. Ginseng Res..

[80] So H (2007). Cisplatin cytotoxicity of auditory cells requires secretions of proinflammatory cytokines via activation of ERK and NF-κB. J. Assoc. Res. Otolaryngol..

[81] Ma H (2010). Cisplatin compromises myocardial contractile function and mitochondrial ultrastructure: Role of endoplasmic reticulum stress. Clin. Exp. Pharmacol. Physiol..

