# Supplementary material for: Sulindac acetohydrazide derivative attenuates against cisplatin induced organ damage by modulation of antioxidant and inflammatory signaling pathways
Source: Sci Rep. 2022 Jul 11;12:11749. doi: 10.1038/s41598-022-15950-9 (PMC9273647; doi:10.1038/s41598-022-15950-9)
Supplement: Supplementary file 1 — Supplementary Information. [file 41598_2022_15950_MOESM1_ESM.pdf]

Supplementary file S1: F1

Sulindac Acetohydrazide derivative attenuates against cisplatin induced organ damage by modulation of antioxidant and inflammatory signaling pathways

Suhail Razak[1#*], Tayyaba Afsar[1#], Nousheen Bibi[2#], Mahmoud Abulmeaty[1], Mashooq Ahmad Bhat[3]*, Anam Inam[2], Janeen H. Trembley[5,6,7], Ali Almajwal[1], Maria Shabbir[4,] Nawaf W. Alruwaili[1], Abdulrahman Algarni[8].

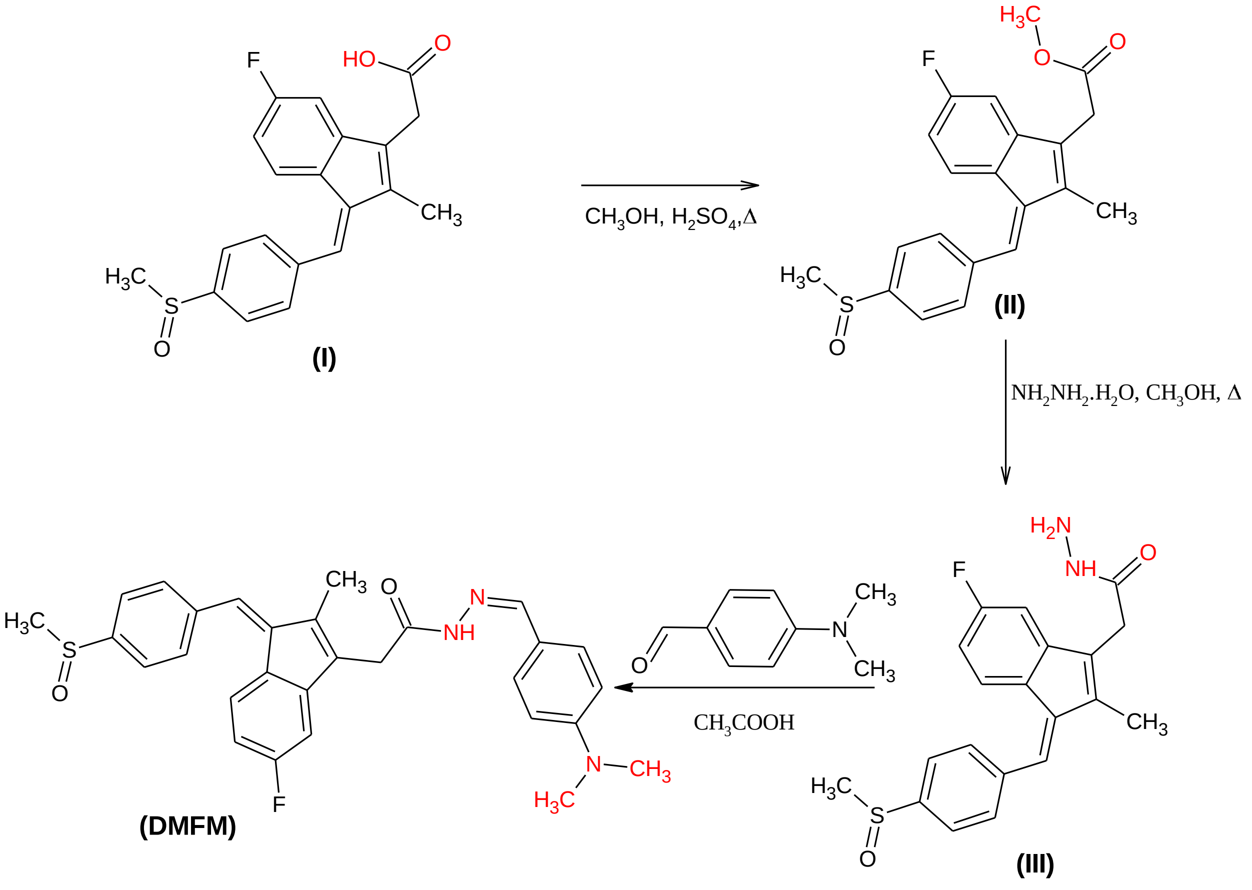

Figure 1: Scheme 1. Synthetic route of compound DMFM**.**

Table 1: Biochemical analysis of liver and kidney function biomarkers in blood serum studied in pilot experiment

	Treatments					
**Parameters**	Saline	CDDP (12.5 mg/kg)	CDDP (12.5 mg/kg) + DMFM (200 mg/kg)	CDDP (12.5 mg/kg) + DMFM (100 mg/kg)	CDDP (12.5 mg/kg) + DMFM (50 mg/kg)	CDDP (12.5 mg/kg) + DMFM (25 mg/kg)
**Total protein (g/dl)**	40.06 ± 0.36	21.25±0.43**	36.67±0.45	35.82±0.38^{++}	35.00±0.32^{++}	34.76±0.32$^{*, ++}$
**ALT (U/l)**	27.15 ± 1.41	65.83 ± 1.12****	32.19±0.51$^{*,++++}$	37.70±0.49$^{**,++++}$	38.03±0.52$^{**,++++}$	39.98 ± 1.52$^{**,++++}$
**ALP (U/l)**	91.07 ± 1.33	180.65± 2.09***	93.09 ± 1.10^{+++}	99.09± 1.24$^{*,+++}$	100.01 ± 1.32$^{*,+++}$	103.11±1.23$^{*,++++}$
**AST (U/l)**	25.67 ± 0.58	140.87±2.00****	29.98 ± 0.39^{+++}	31.87 ±0.47 $^{*,++++}$	33.87±0.42$^{*,++++}$	35.79 ±0.45$^{*,++++}$
**Serum creatinine (mg/dl)**	15.11 ± 0.43	83.55±0.99****	20.09± 0.31 $^{*,++++}$	25.15 ± 0.29$^{**,++++}$	28.12 ± 0.41$^{**,++++}$	29.01 ± 0.39$^{**,++++}$
**Urea (mg/dl)**	24.98 ± 0.49	86.97±1.01	28.11 ± 0.30 $^{++++}$	30.22± 0.35$^{**,++++}$	32.09± 0.16$^{**,++++}$	35.01± 0.26$^{**,++++}$

Effect of CDDP on liver function biomarkers (ALT, ALP and AST) indicated significant upsurge in tested parameters. DMFM at all tested doses significantly protected the abnormal increase in serum ALT, AST and ALP levels. Moreover, Cisplatin (CDDP,12.5mg/kg, i.p.) induced significant elevation ($P > 0.001$) in urea and creatinine level after 72 h of administration in comparison to saline group. DMFM administration prior to CDDP inoculation at various dose (200,100, 50 and 25 mg/kg b.w) significantly ($p<0.001$) lower the upsurge of biomarkers in serum. The level of various biomarkers in DMFM treatment groups was noted to be balanced up to normalize the levels. Lower tested dose (25mg/kg b.w) showed significant efficiency against CDDP as highest tested doses. Values expressed as mean ± SEM (n=3). $^{*, **, ****}$ indicates significant difference at $p < 0.05$, $p < 0.001$ and $p < 0.0001$ from saline group whereas ++, ++++

indicates significant difference at $p < 0.001$ and $p < 0.0001$ from CDDP (125 mg/kg b.w) group. (One-way ANOVA followed by Tukey's multiple comparison tests).

Supplementary file S1: Raw western blot data

Liver

Western blot images

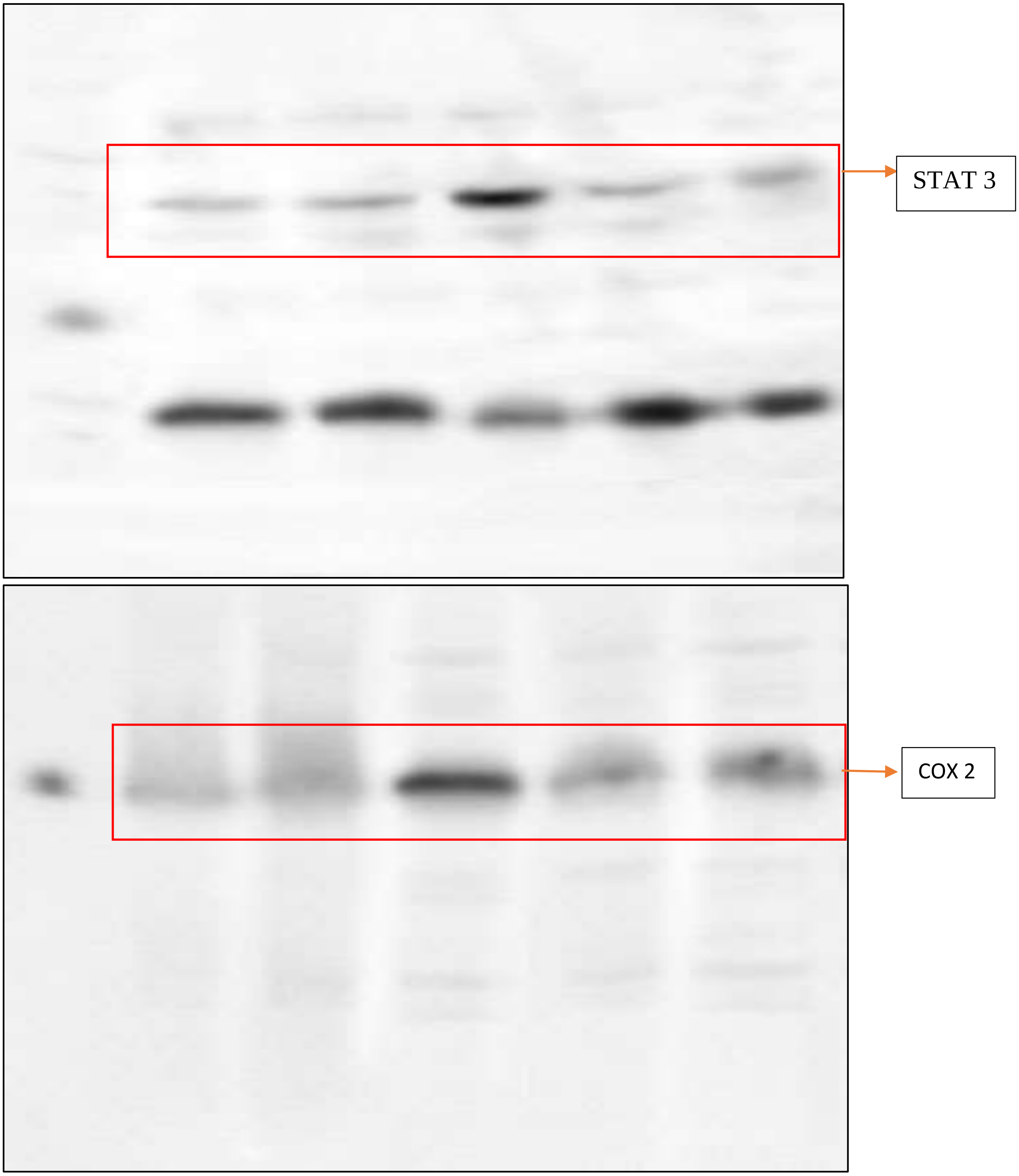
STAT 3
COX 2

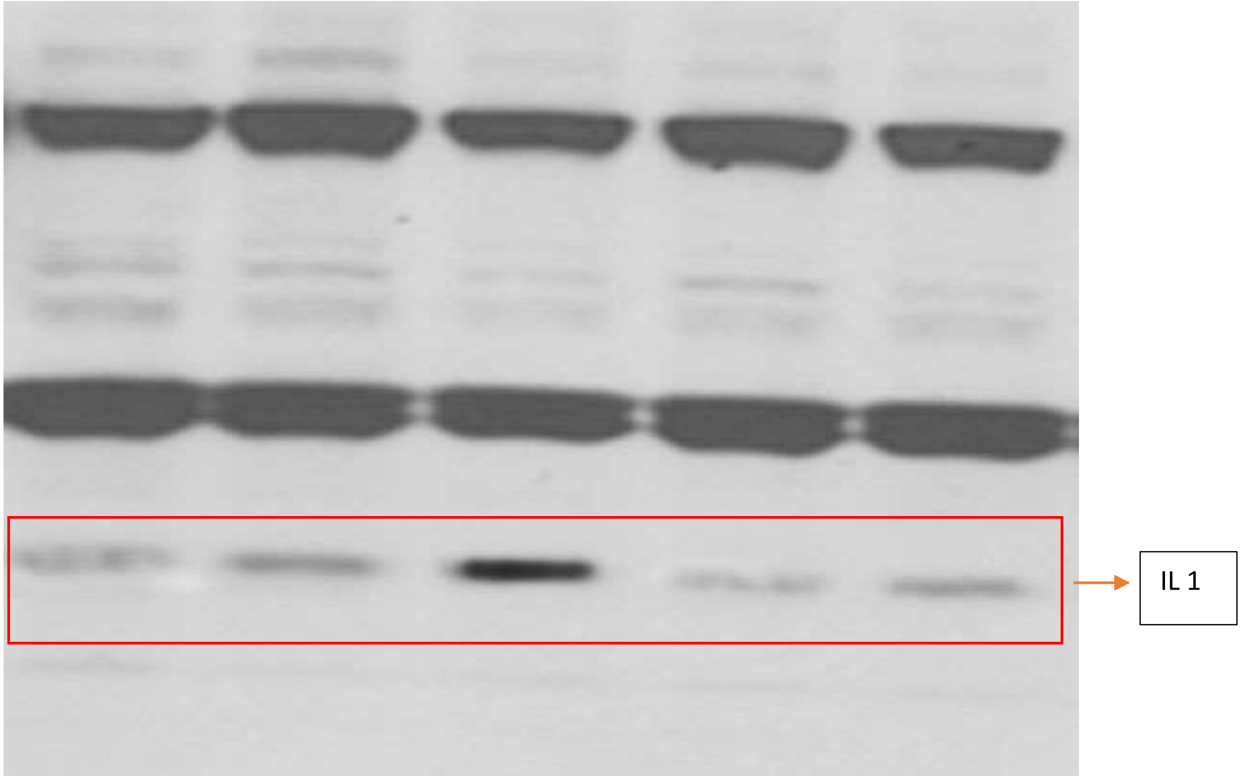
IL 1

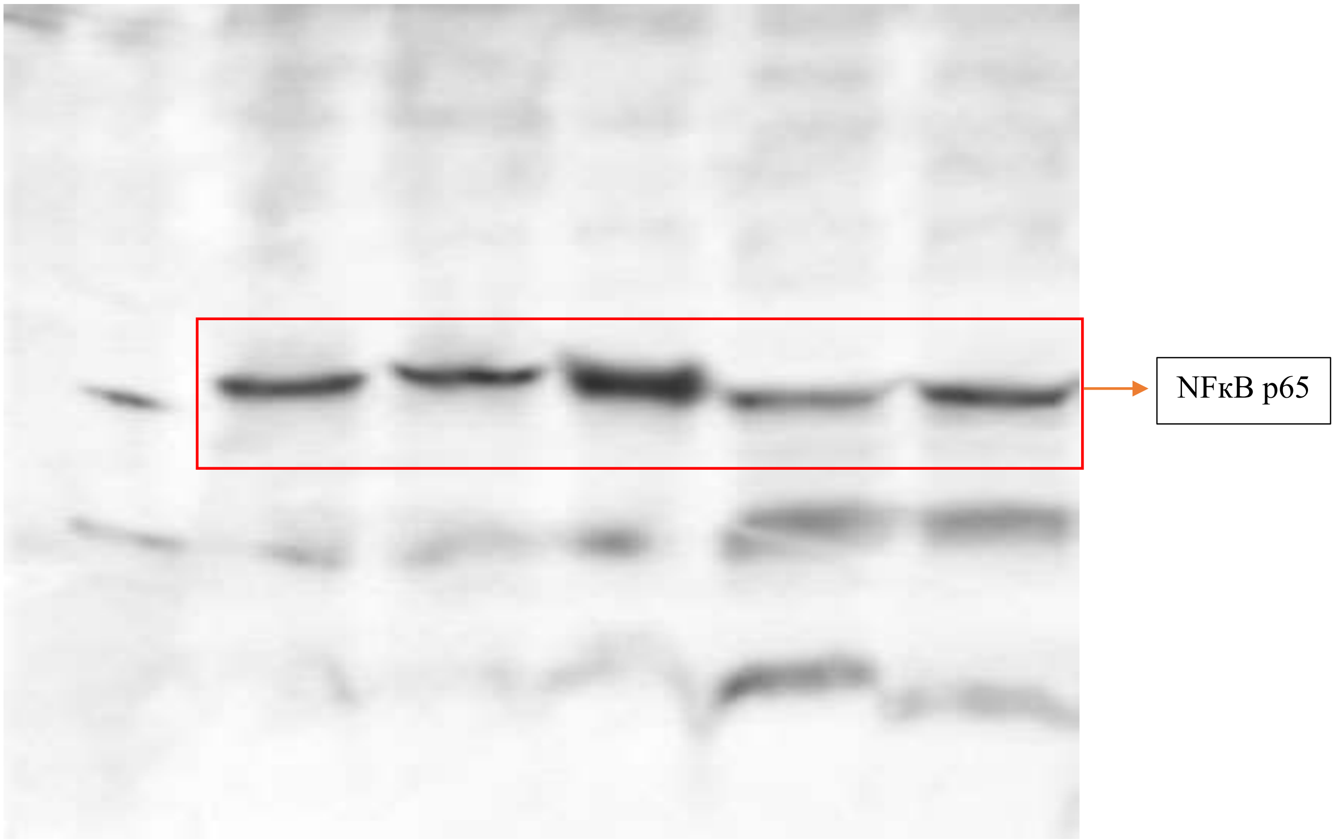
NFκB p65

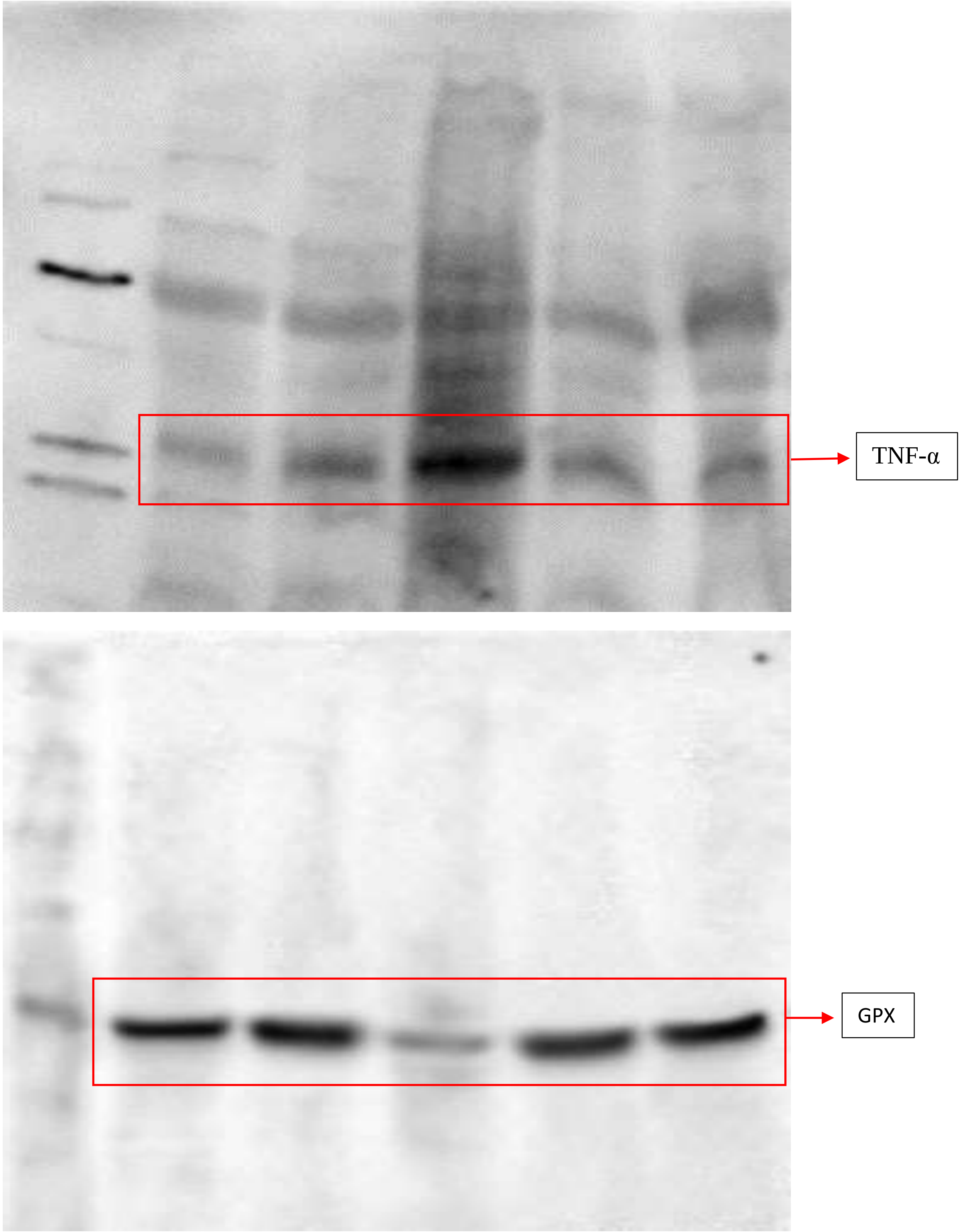
TNF-α
GPX

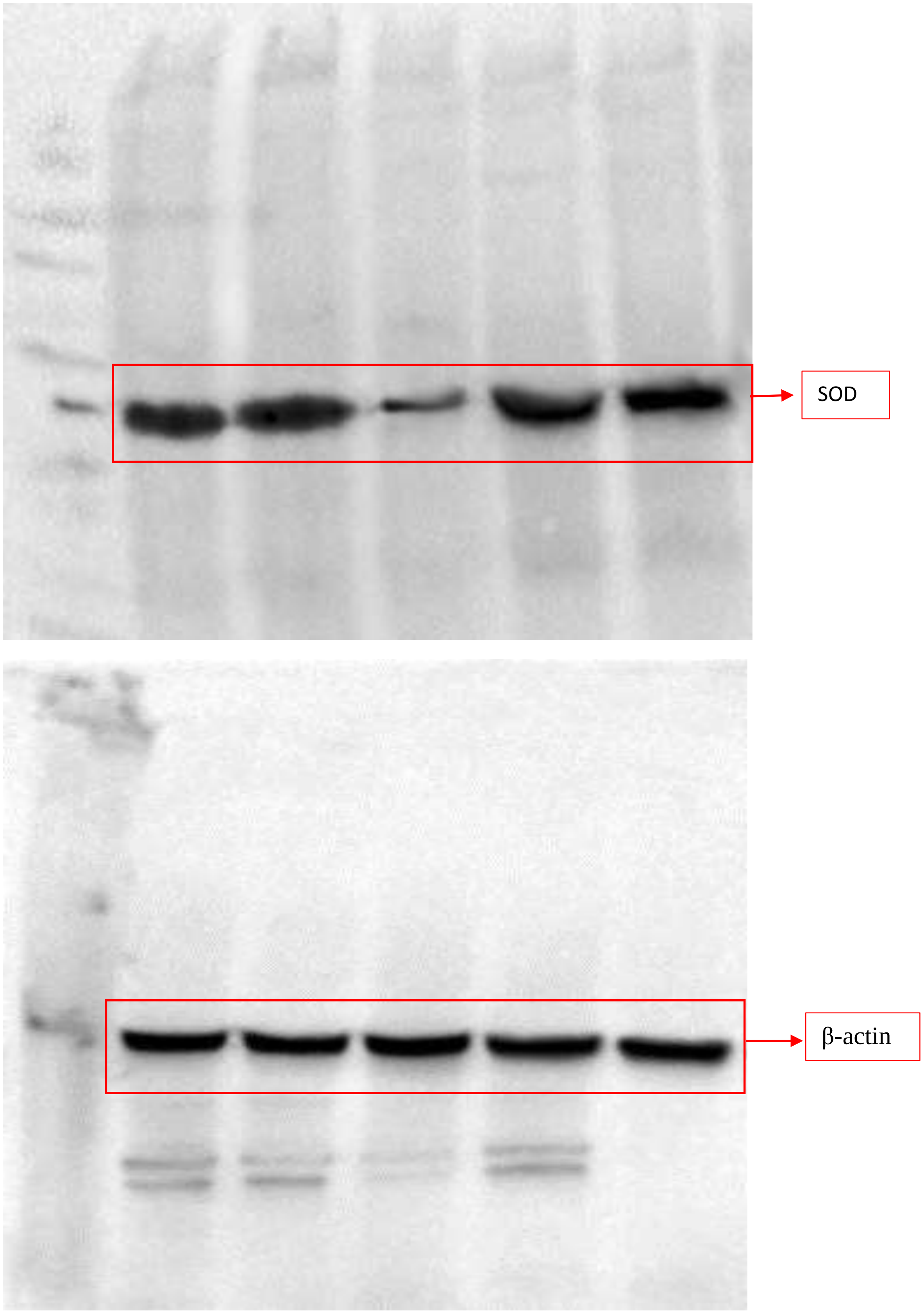
SOD
β-actin

Liver cropped images

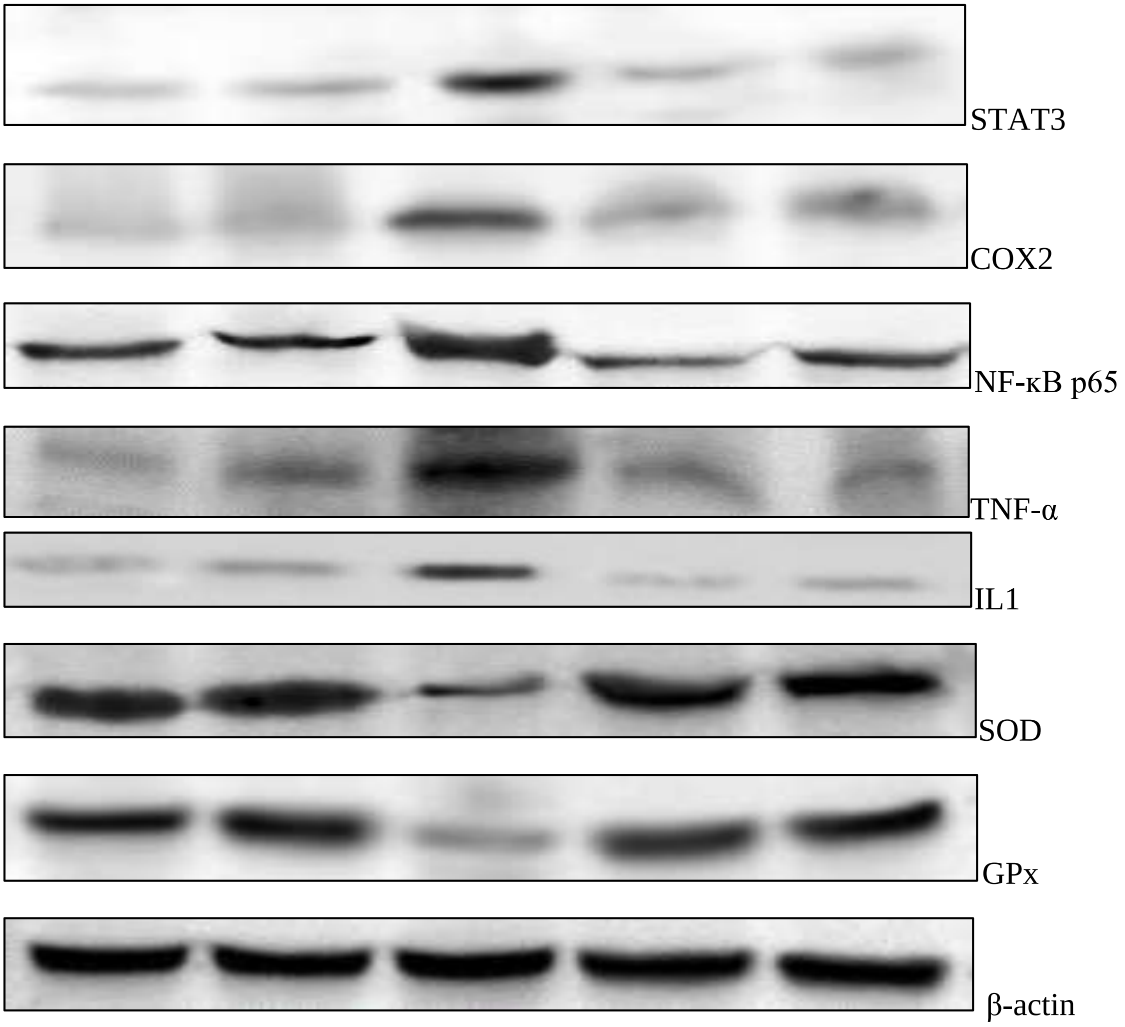

Heart

western blot images

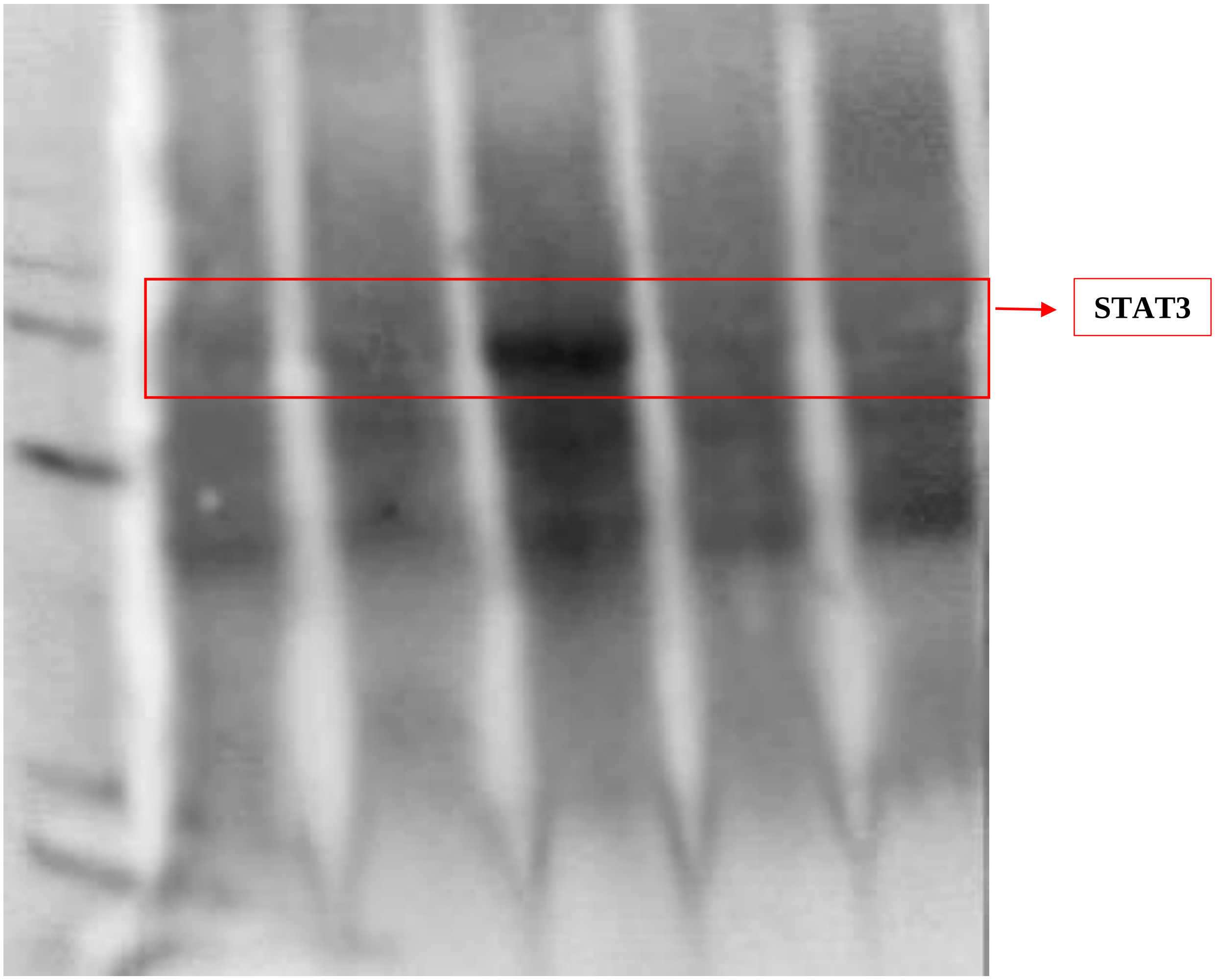

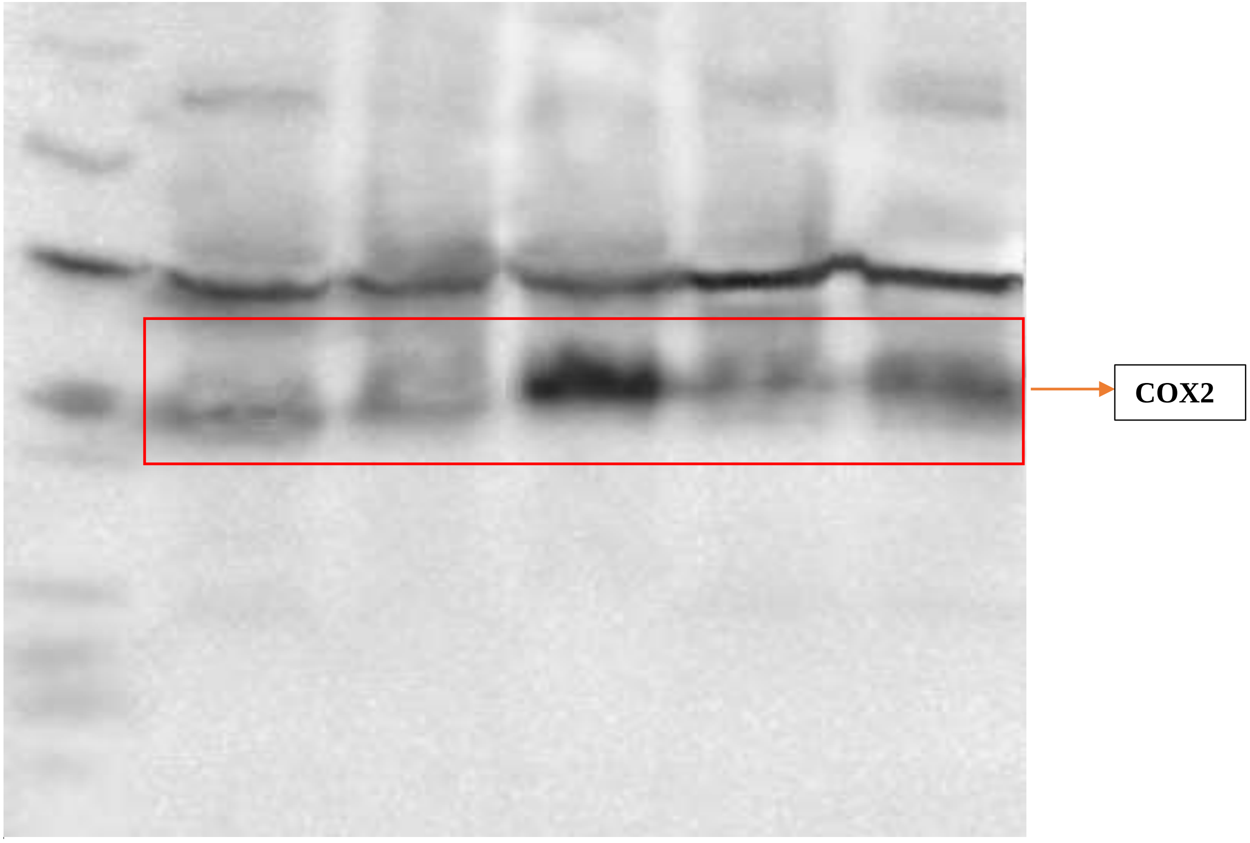
COX2

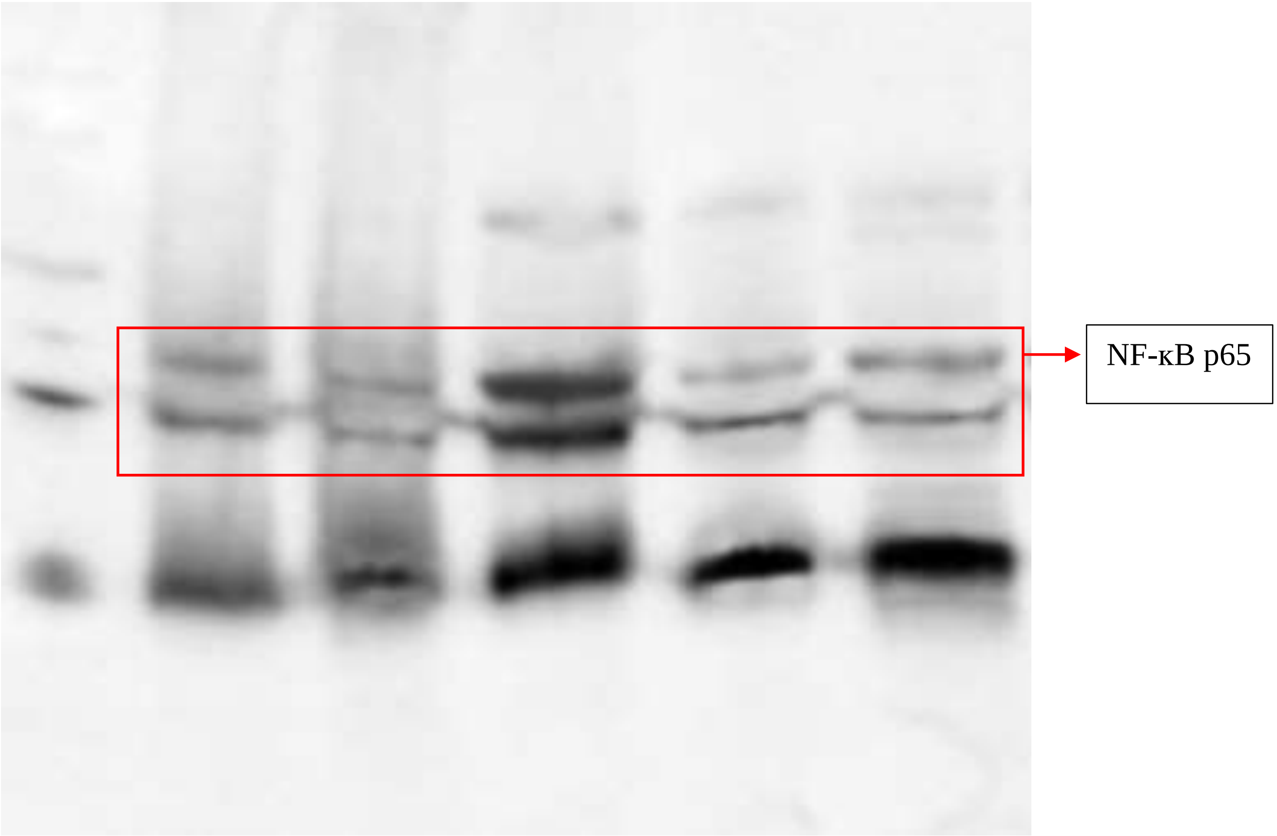
NF-κB p65

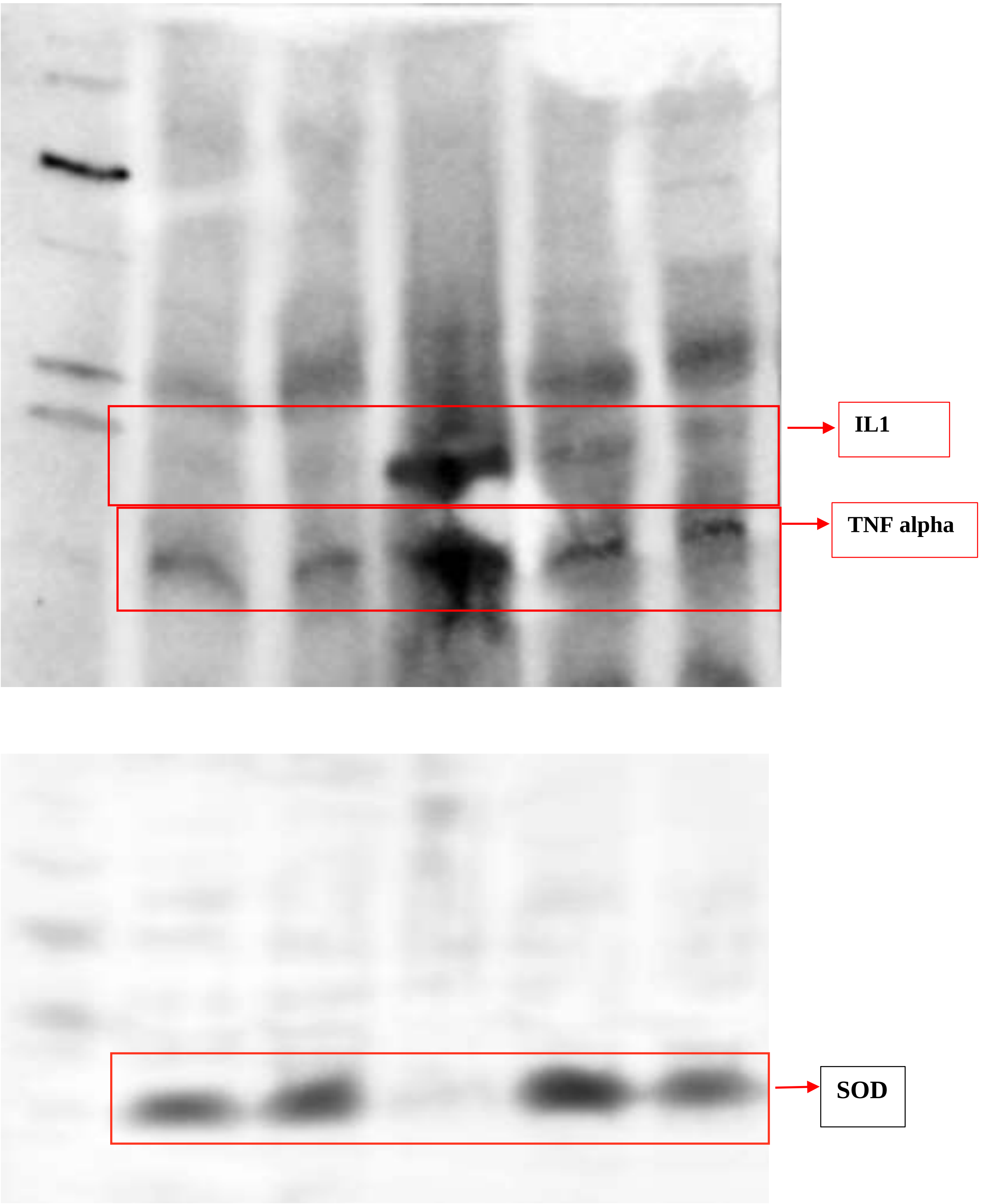
IL1
TNF alpha
SOD

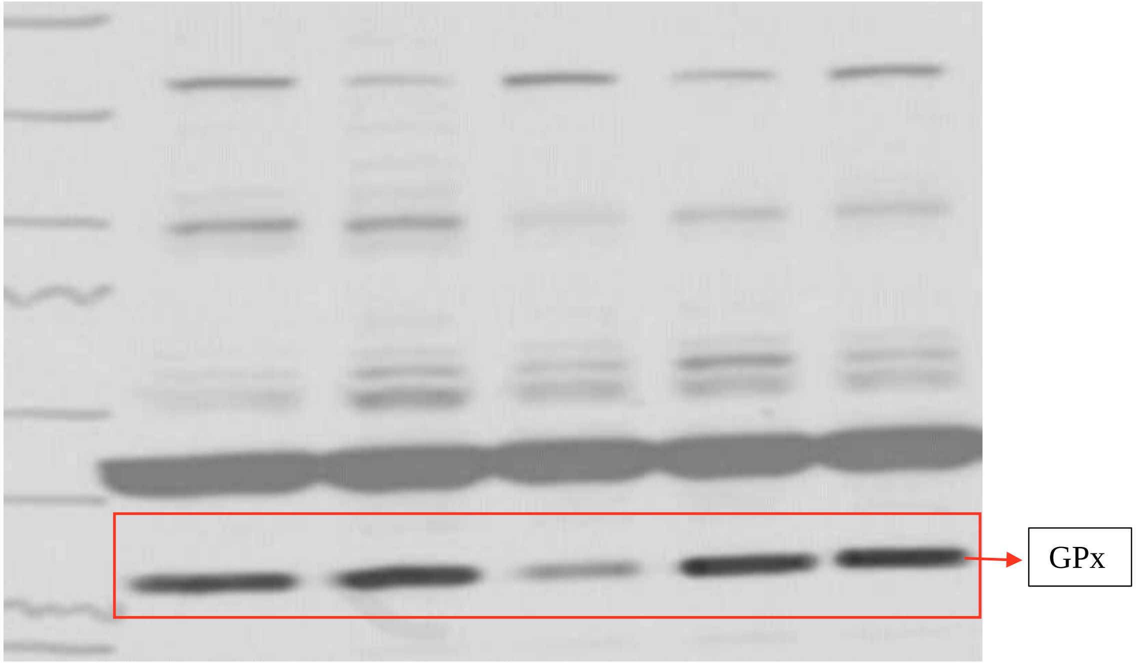
GPx

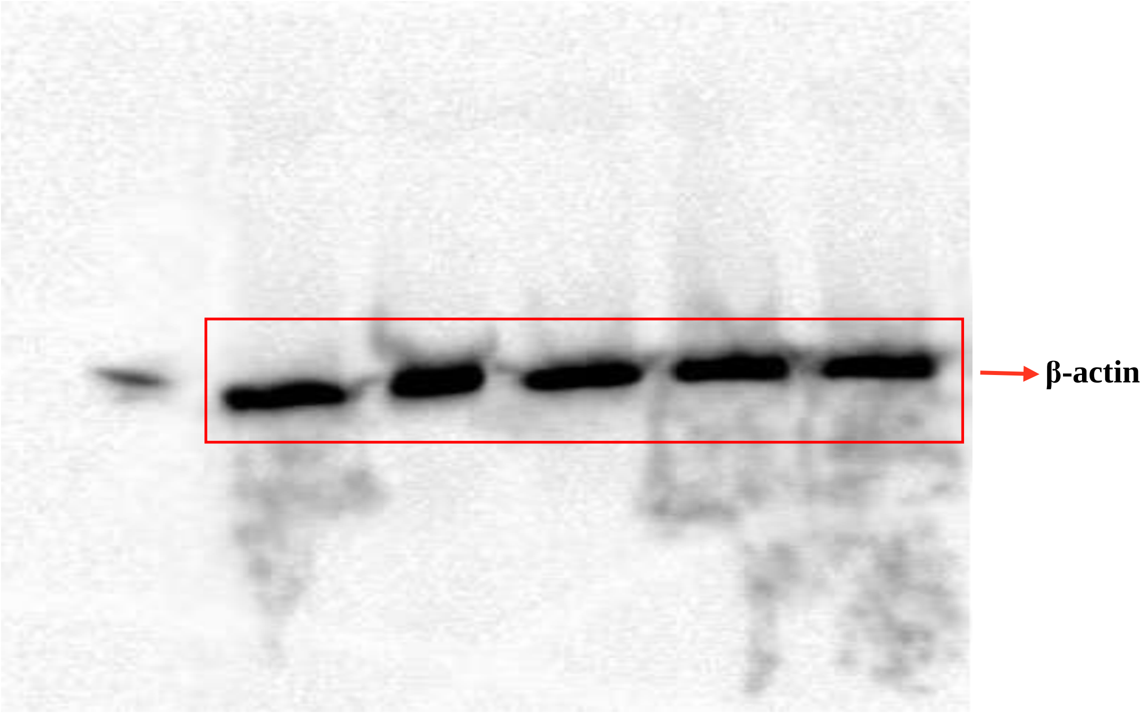
β-actin

Cropped images heart

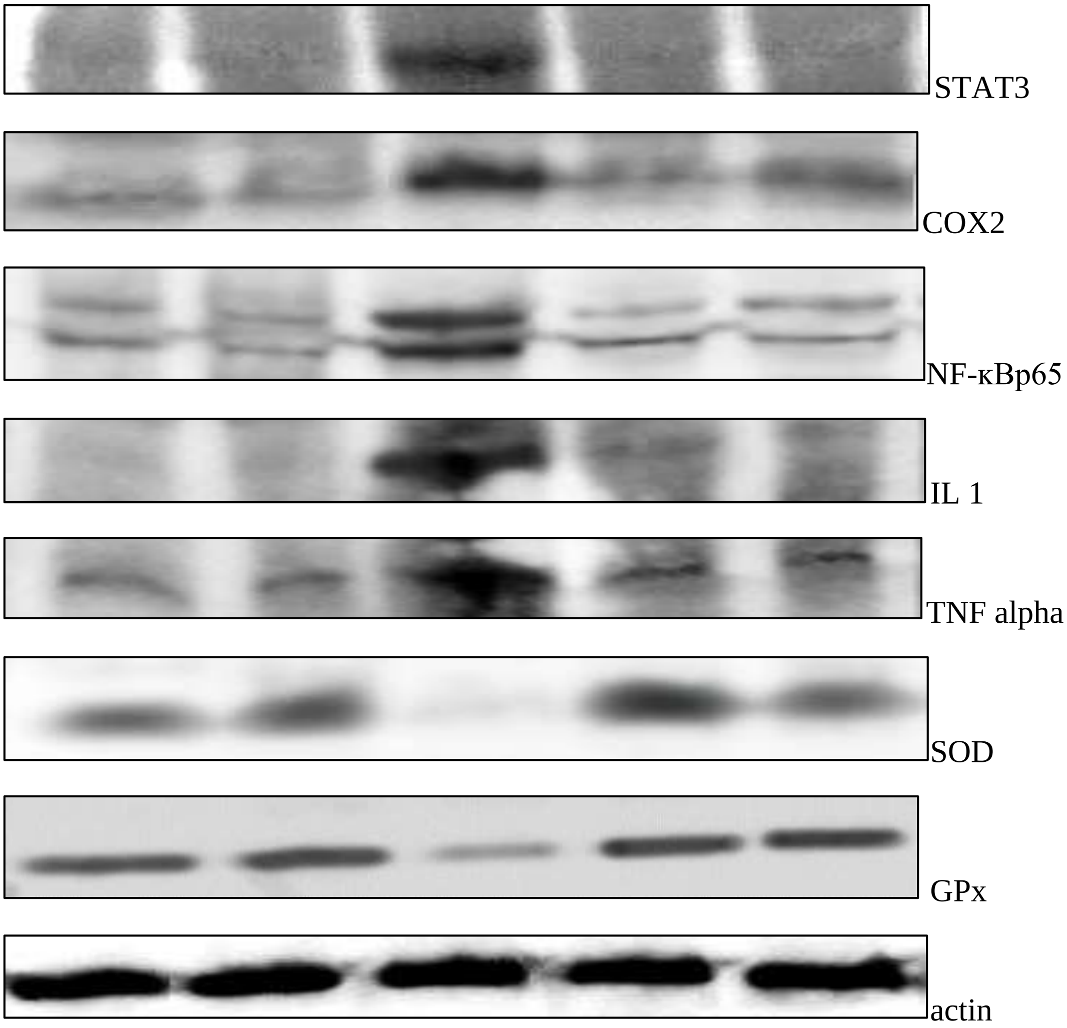

Kidney

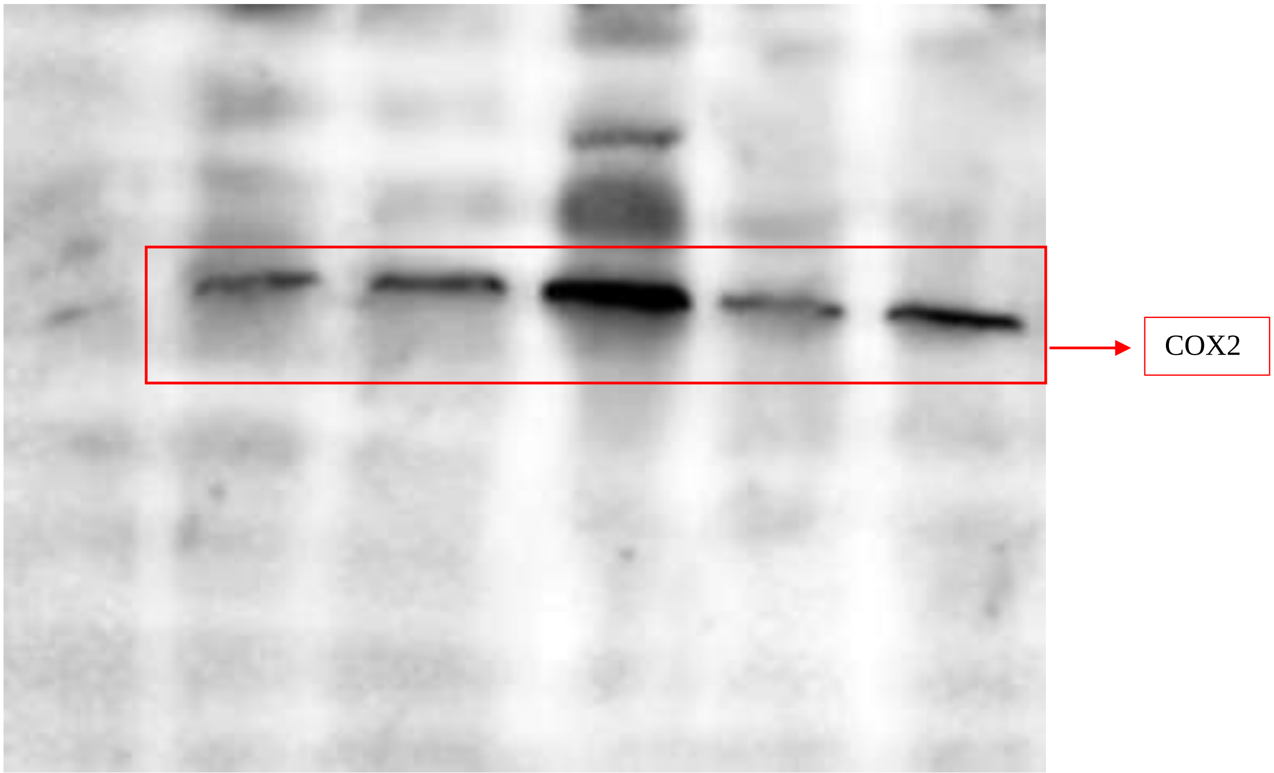

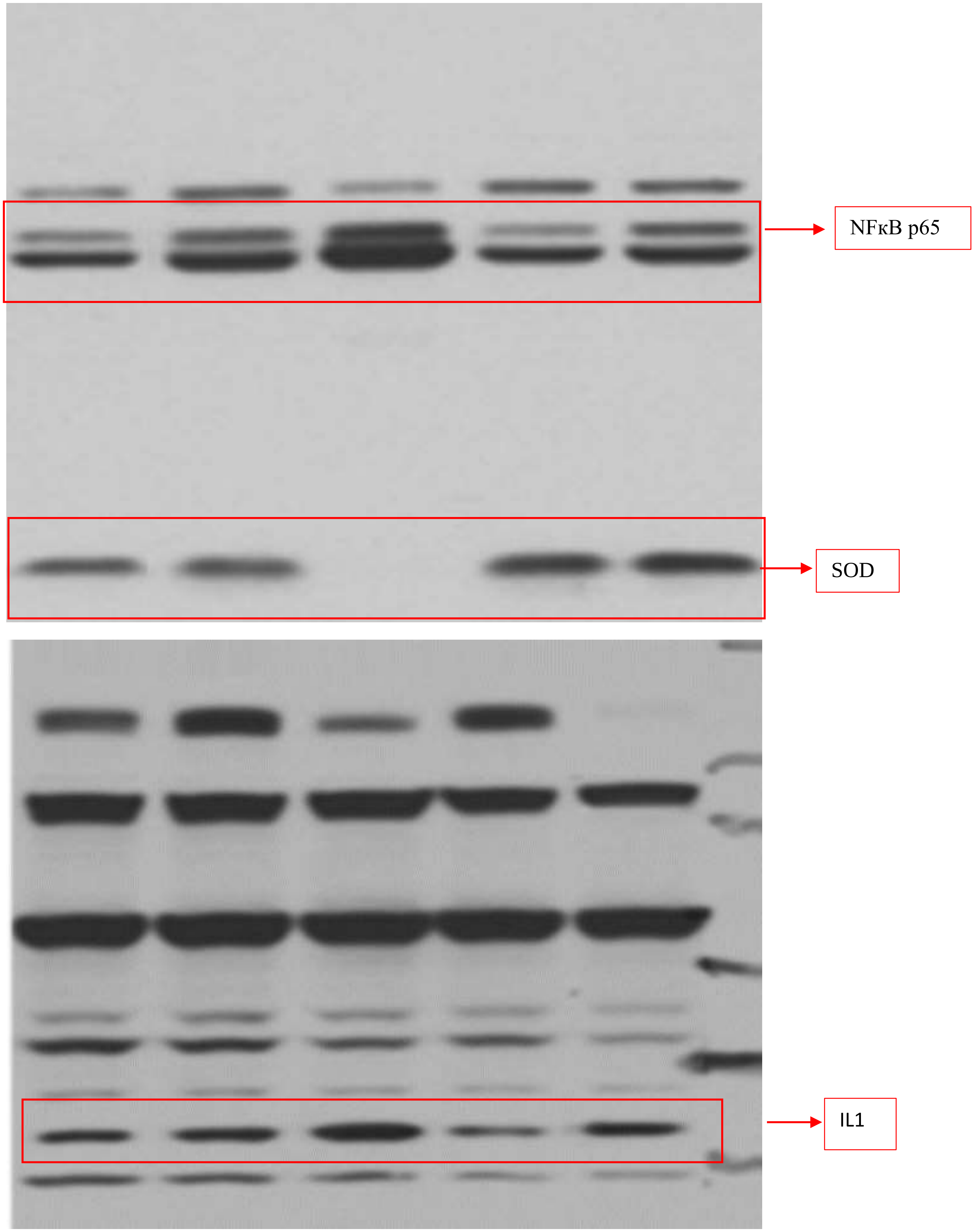
NFκB p65
SOD
IL1

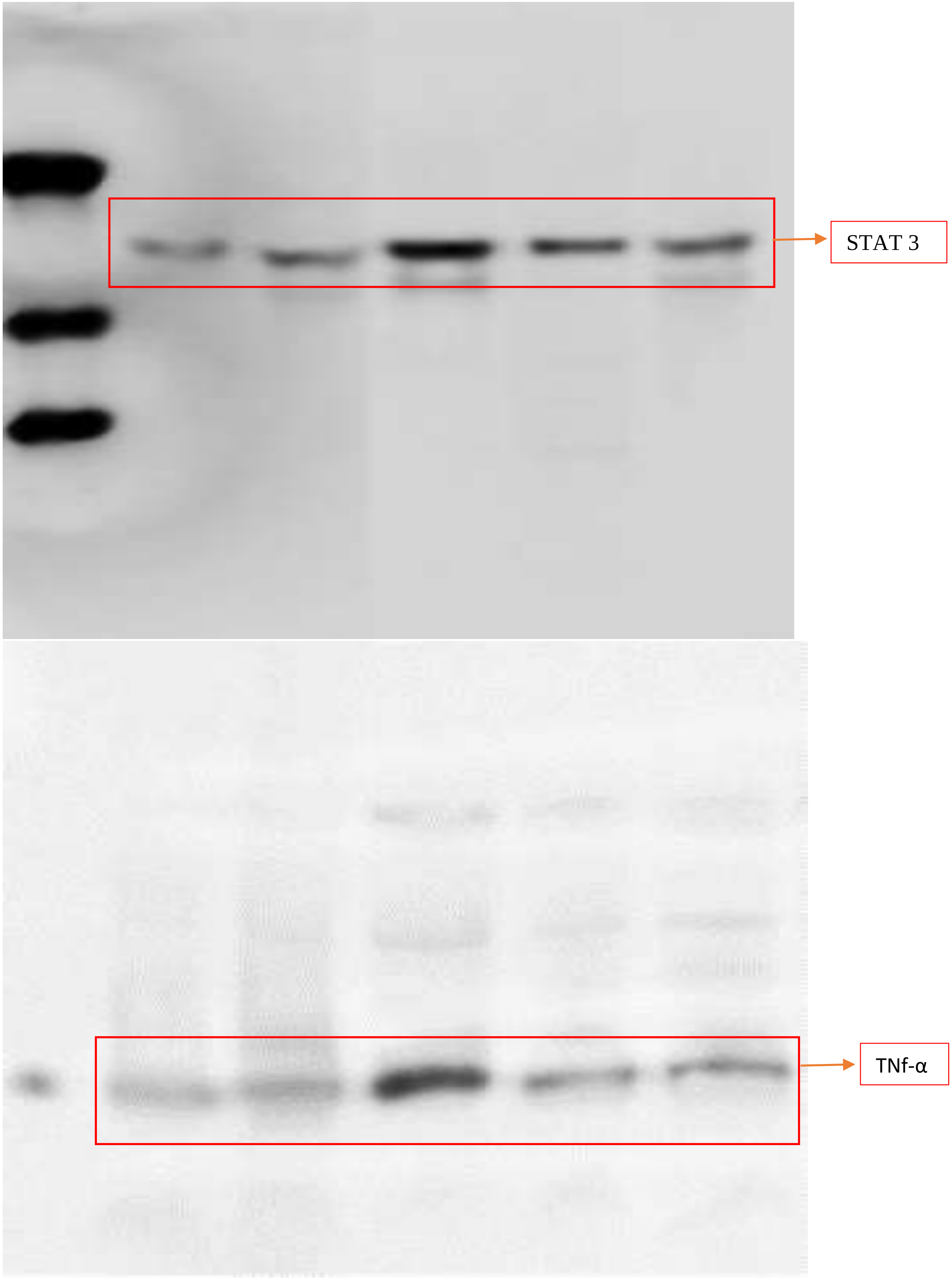
STAT 3
TNf-α

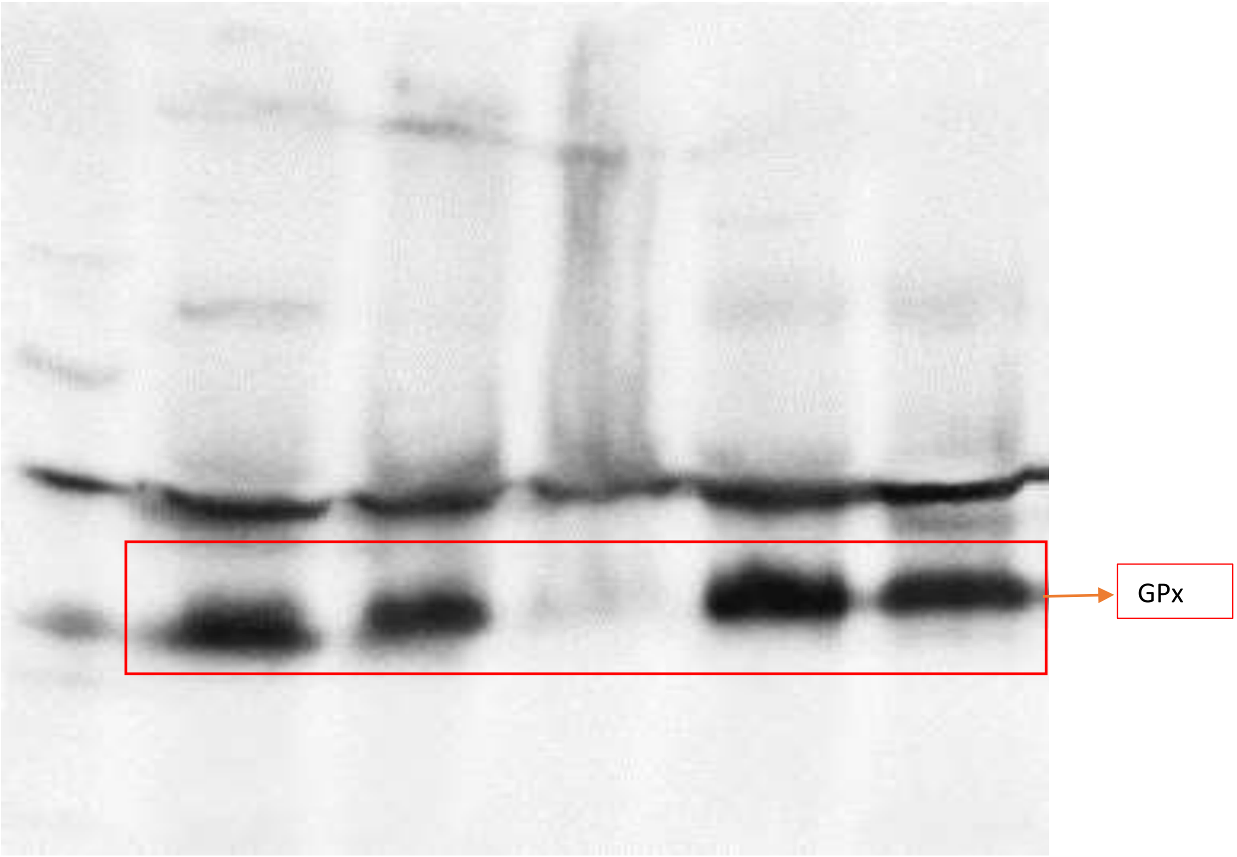
GPx

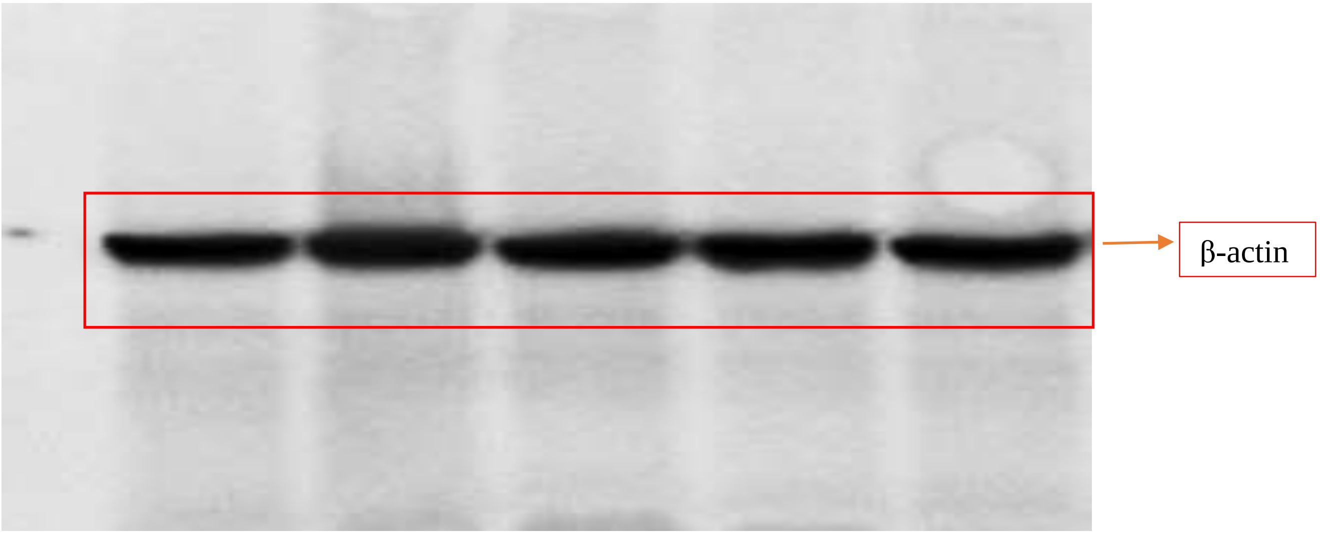
β-actin

Cropped images kidney

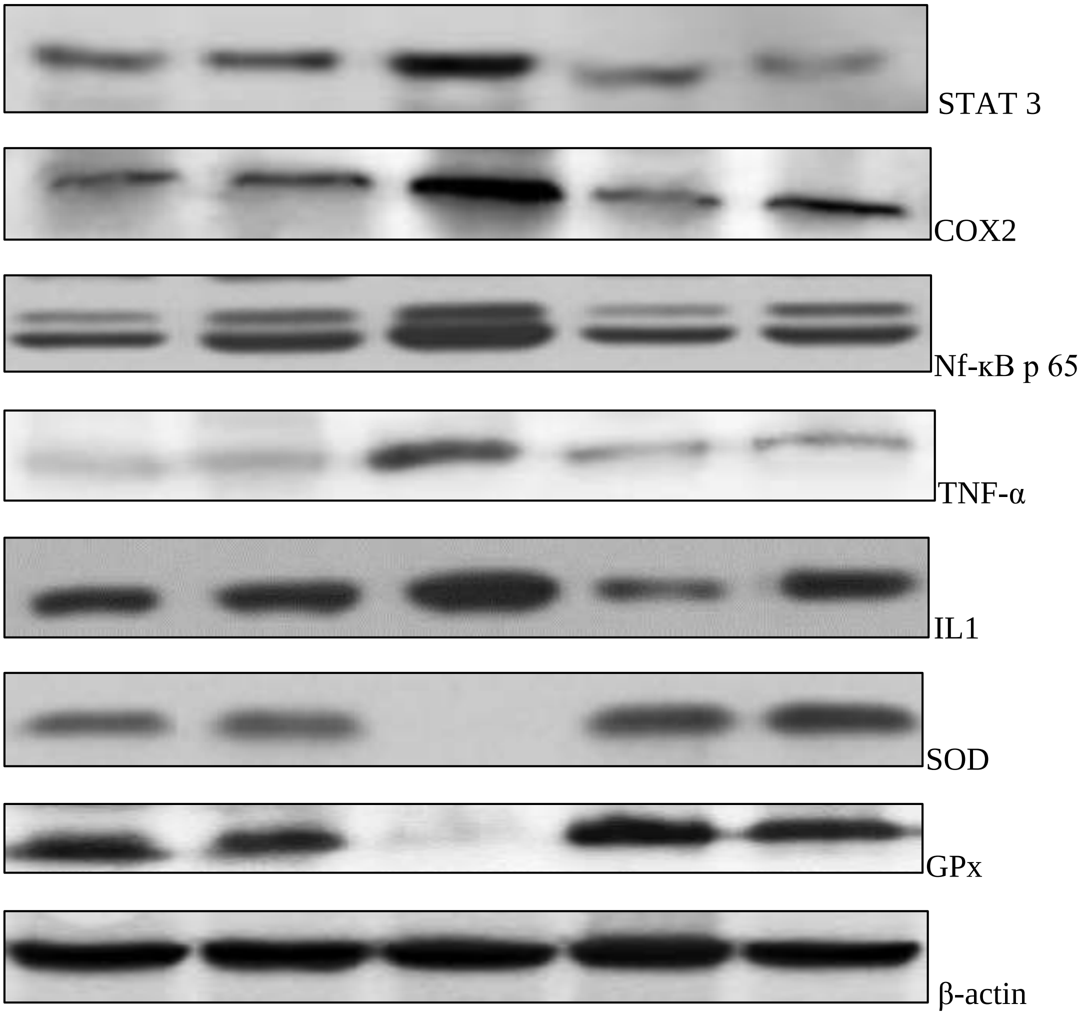

Brain

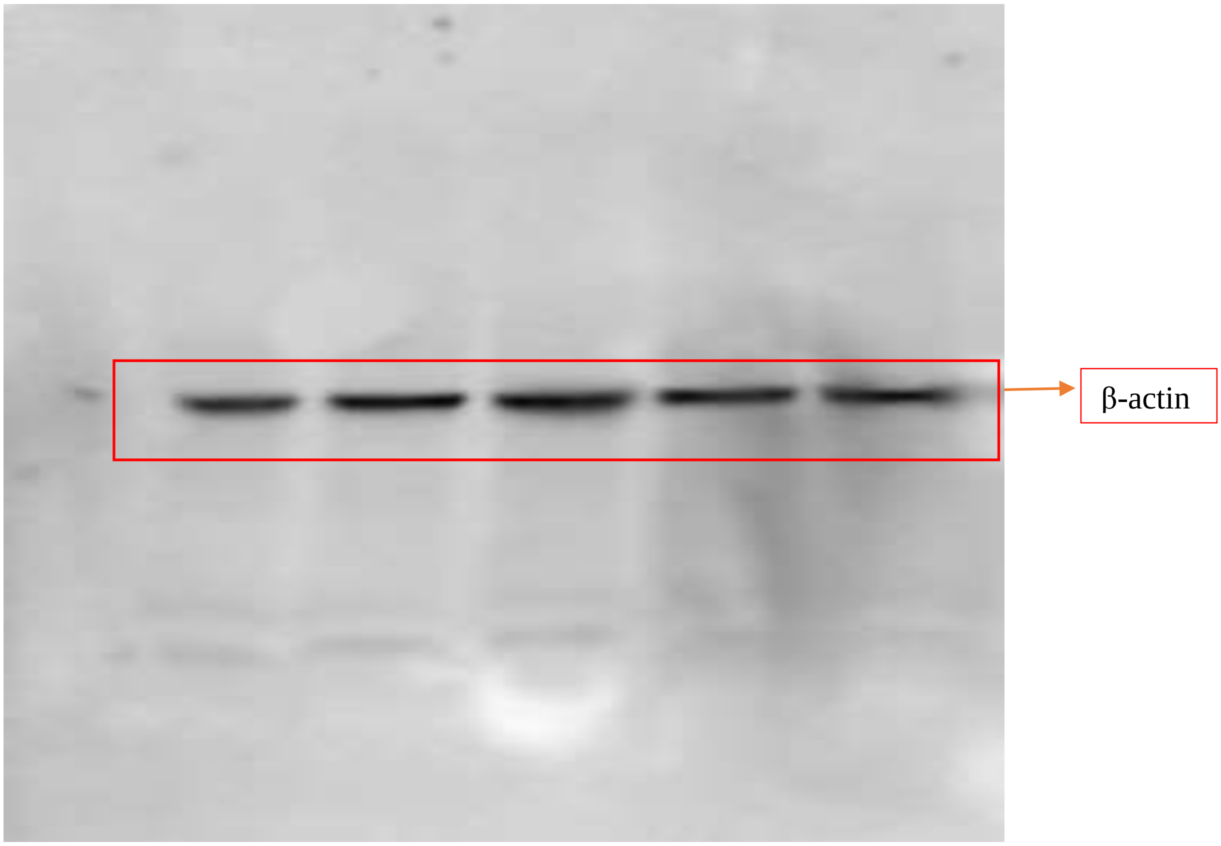

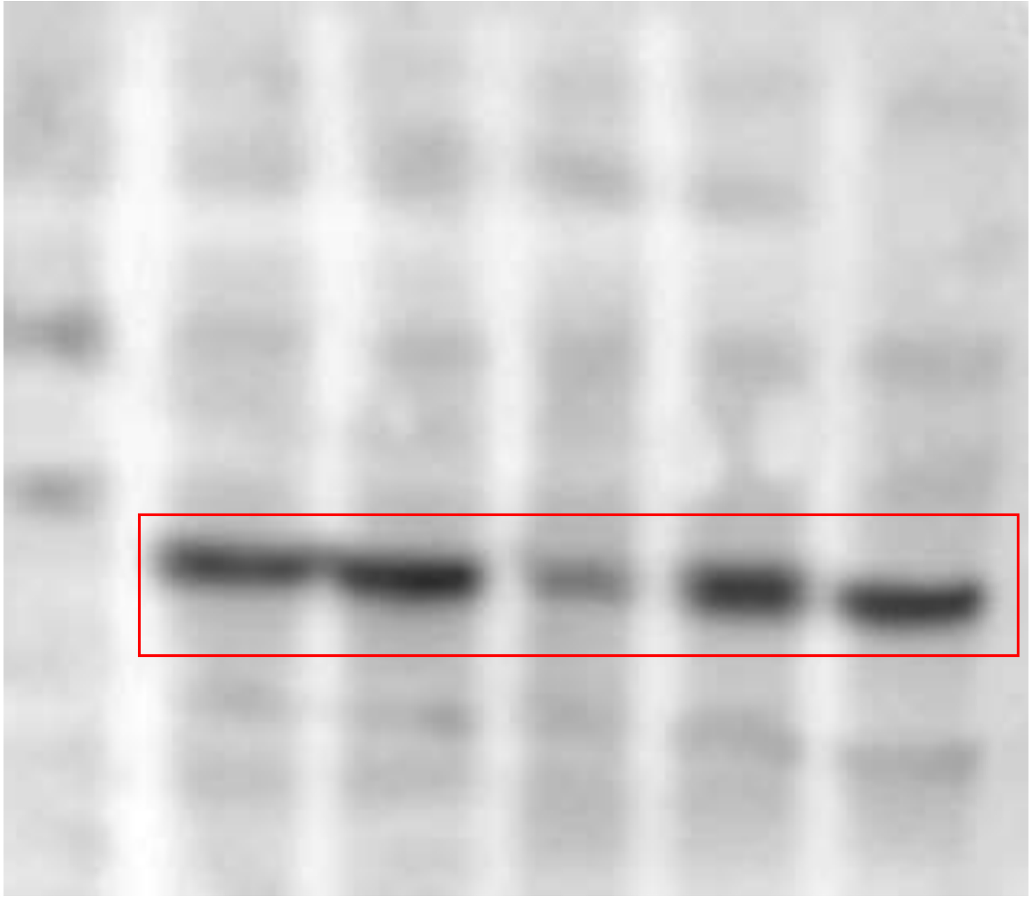

SOD

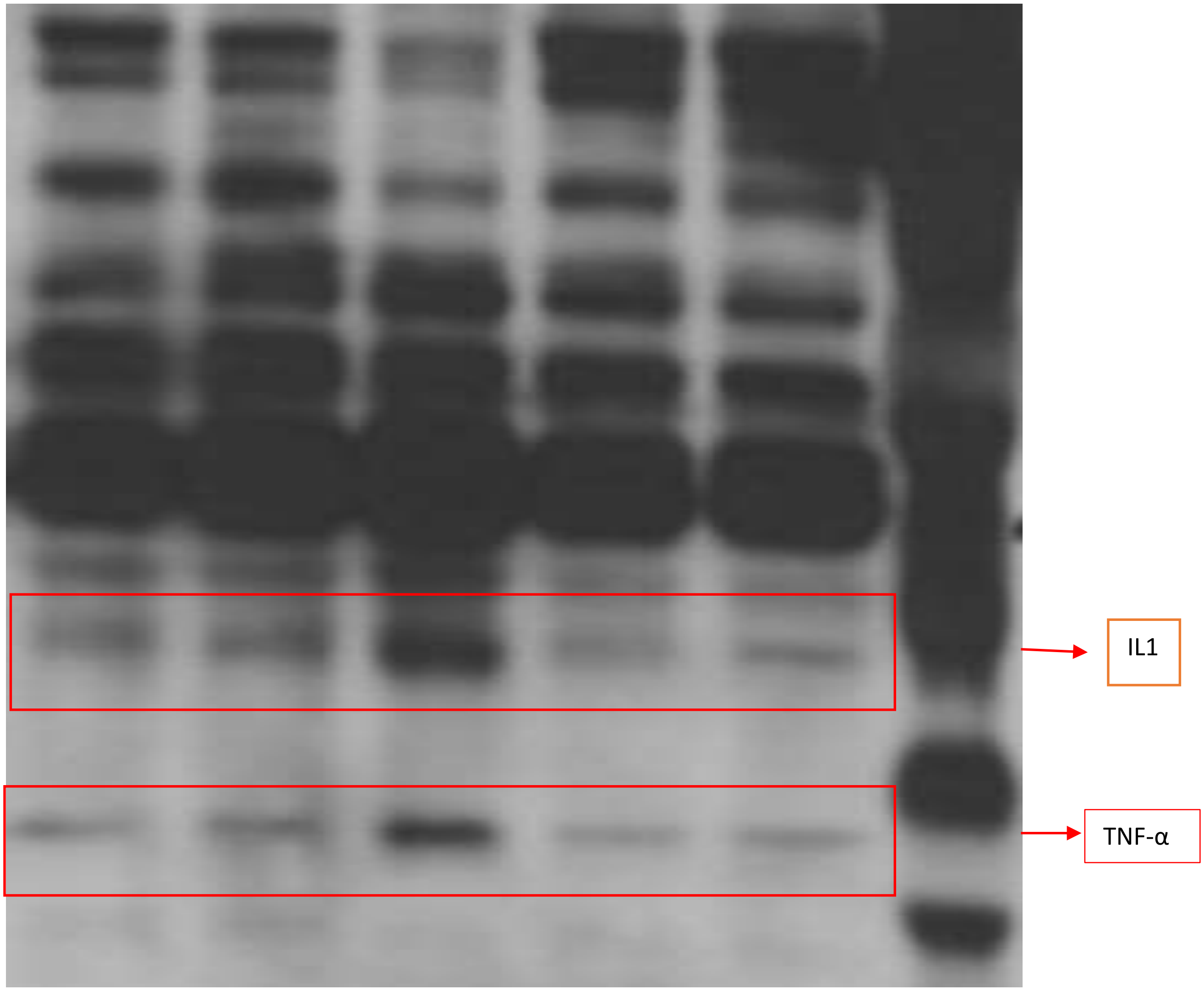
IL1
TNF-α

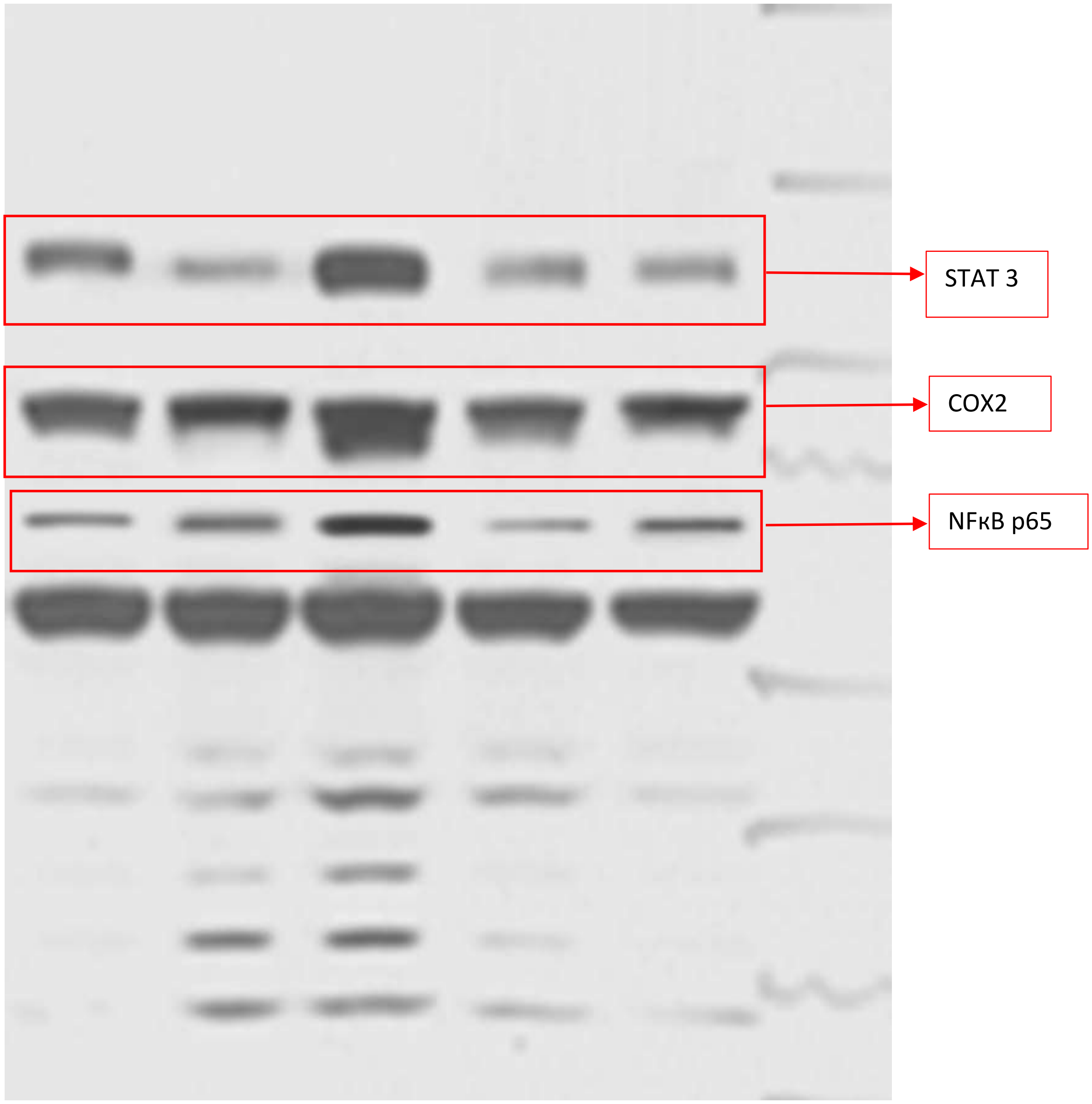
STAT 3
COX2
NFκB p65

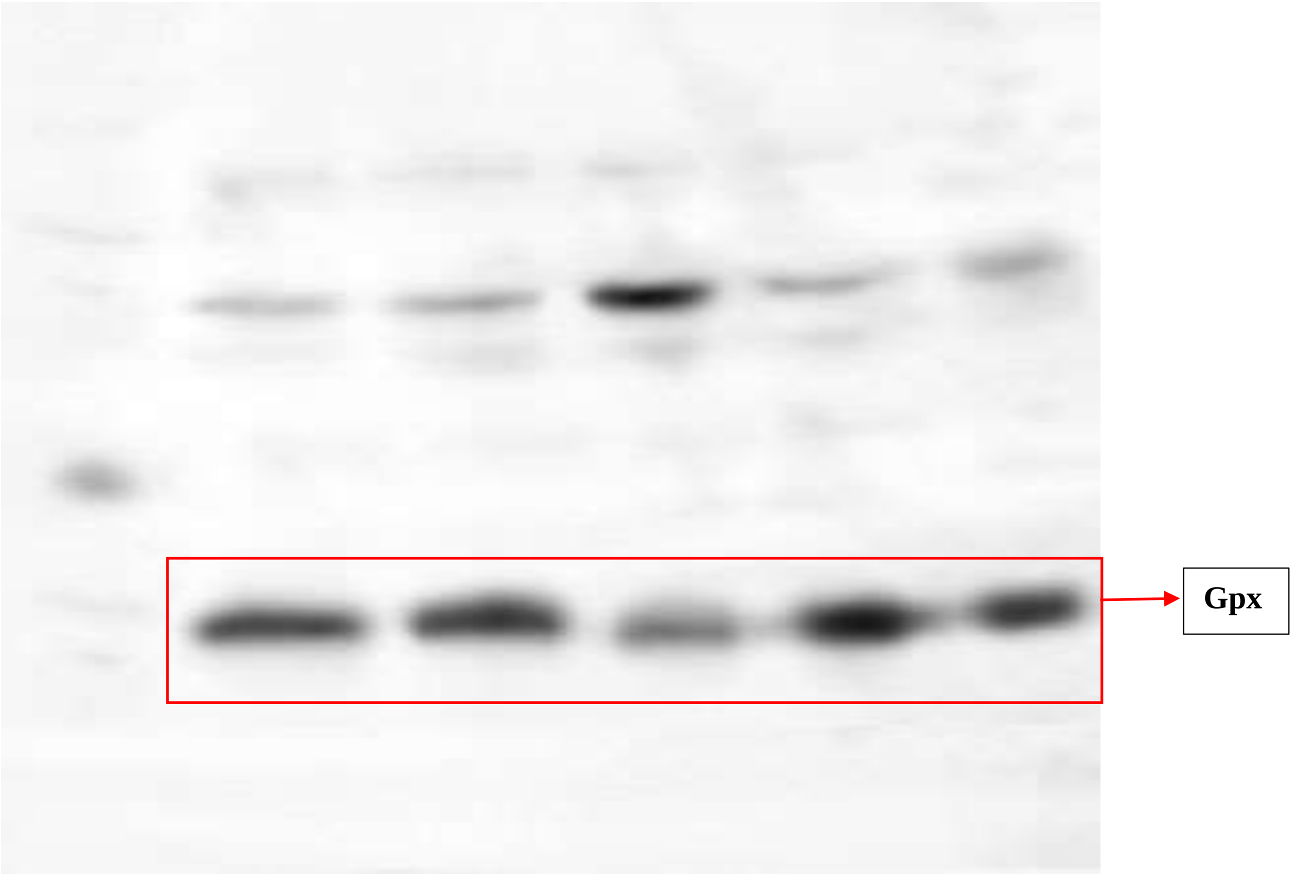

Cropped blots brain

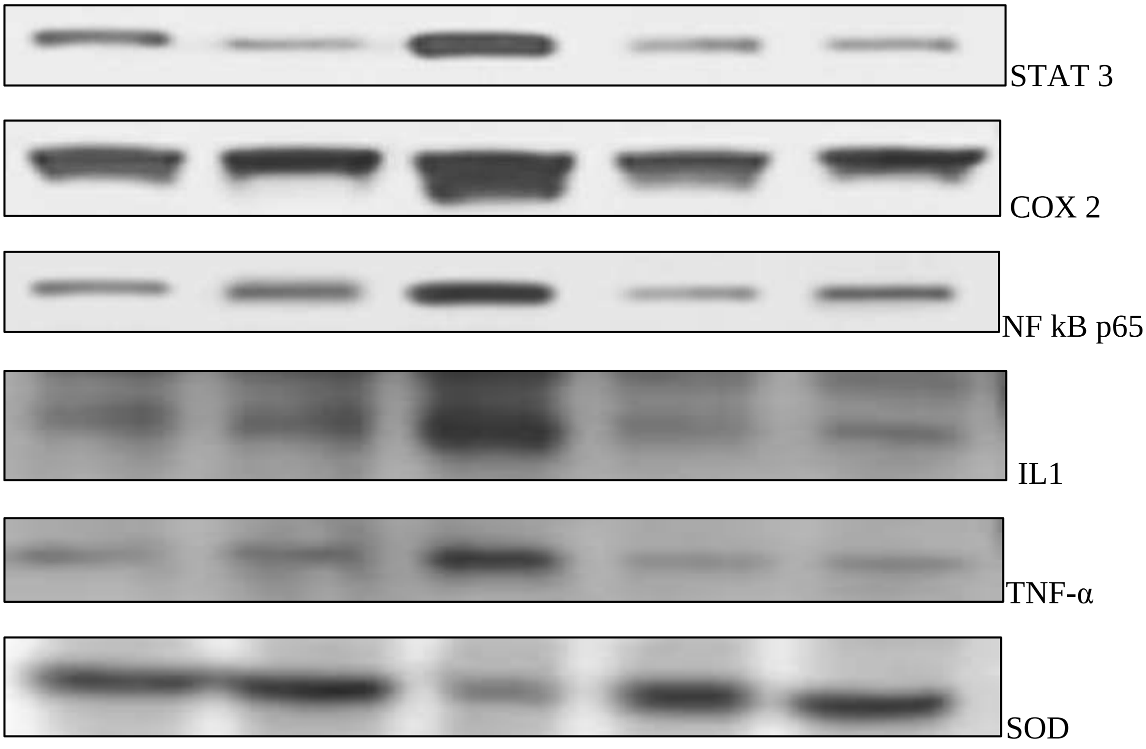

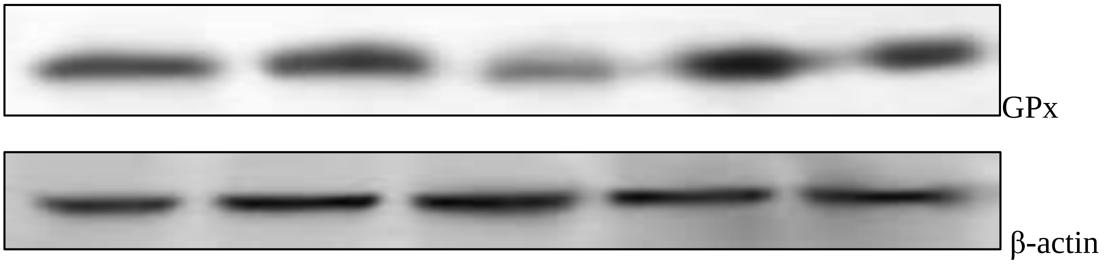

Figure2 S1: Raw data of cropped gels and cropped
